# Structural and functional insights into *Listeriamonocytogenes* phage endolysin PlyP100: A promising food safety tool

**DOI:** 10.1016/j.jbc.2025.110295

**Published:** 2025-05-27

**Authors:** Karson R. Bateman, Emma Scaletti Hutchinson, Göran Widmalm, Michael J. Miller, Pål Stenmark

**Affiliations:** 1 Department of Food Science and Human Nutrition, University of Illinois Urbana-Champaign, Urbana, Illinois, USA; 2 Department of Biochemistry and Biophysics, Stockholm University, Stockholm, Sweden; 3 Department of Chemistry, Stockholm University, Stockholm, Sweden

**Keywords:** queso fresco, *Listeria monocytogenes*, endolysin, *Listeria* phage, modularendolysins

## Abstract

*Listeria monocytogenes* is a ubiquitous, psychrotrophic human pathogen that can cause listeriosis, a serious illness for vulnerable populations. Some foods, such as Hispanic-style fresh cheeses like queso fresco, pose a specific risk because there are no widely accepted or available methods for *L. monocytogenes* mitigation that are both effective and able to maintain the properties of the products. *Listeria-*specific bacteriophages encode endolysins that can cleave the peptidoglycan layer of *L. monocytogenes* cells externally, showing promise as a potential solution to this problem. PlyP100, from the GRAS *Listeria* phage P100, is one such endolysin that can prevent the growth of *L. monocytogenes* in both lab culture conditions and a miniaturized queso fresco model. In this work, we aimed to understand the structural and functional properties of PlyP100. An AlphaFold prediction suggested the presence of three separate domains (D1, D2, and D3). By solving the crystal structure of D1 and assessing various domain truncations, we present evidence that D1 is responsible for catalytic activity, D3 is sufficient for cell wall binding, and D2 is necessary for full function of the enzyme against live cells. Additionally, we performed point mutations in D1 and compared PlyP100 to proteins with similar structures, including *Streptococcus pneumoniae* LytA and *Listeria* endolysin Ply511, to understand its specific enzymatic mechanism and target strain specificity. These insights into the structure and function of PlyP100 will aid future work aiming to engineer better endolysins as safe food antimicrobials.

*Listeria monocytogenes* is a Gram-positive species of bacteria responsible for causing listeriosis, a relatively uncommon but disproportionately severe foodborne illness (1). Listeriosis can cause severe illness including sepsis, meningitis, and even death in vulnerable populations, namely, fetuses, infants, and people who are pregnant, elderly, or immunocompromised (2). *L. monocytogenes* is environmentally prevalent, and pasteurization and cooking methods are used to eliminate the pathogen. However, *Listeria* spp. are universal and psychrotrophic, so if food products are contaminated after pasteurization, *L. monocytogenes* growth is not prevented by refrigeration temperatures (3). This makes ready-to-eat (RTE) foods such as deli meat, hot dogs, and soft/fresh cheeses a specific concern (4).

Queso fresco (QF) is a soft, high-moisture, unripened Hispanic-style cheese that has been rising in popularity in the United States (5). The neutral pH and sub-inhibitory salt content of QF and similar products provide favorable conditions for *L. monocytogenes* growth. Currently, there are no widely accepted or available methods for *Listeria* mitigation in fresh cheeses that are both effective and able to maintain the sensory properties of the products (6). *Listeria-*specific bacteriophage endolysins have been proposed as a promising solution to address this problem (7, 8, 9). Endolysins are expressed by bacteriophages at the end of their lytic cycle to hydrolyze the peptidoglycan (PG) layer of their host cell. While intended for “lysis-from-within,” it has been demonstrated that endolysins can also effectively lyse PG and kill target Gram-positive bacteria from outside of the cell (10). Taking advantage of this external lysis activity may be a solution to the ongoing problem of improving the safety of QF and other fresh cheeses, without compromising the sensory qualities of the product.

In the cell envelope of Gram-positive bacteria, the thick PG network (11, 12) forms a mesh-like structure that contains polysaccharides with disaccharide repeating units, *i.e*., [→4)-β-MurNAc-(1 → 4)-β-d-GlcNAc-(1→]_n_. These polysaccharides are substituted at the MurNAc residues by short peptides, and in *L. monocytogenes*, whose PG is of the A1γ type, they are directly crosslinked (13, 14) (Fig. S1). Our current research focuses on the *Listeria-*specific *N*-acetylmuramoyl-l-alanine amidase PlyP100, an endolysin encoded by the bacteriophage P100, which cleaves PG at the amide linkage between the first l-alanine in the peptide chain and the lactyl group of MurNAc. Previous studies have shown that PlyP100 has a listeriastatic effect in biologically relevant conditions, making it a promising tool for preventing *L. monocytogenes* growth in fresh cheeses (7, 15, 16, 17). Additionally, its parent phage, P100, has already been approved as Generally Recognized As Safe (GRAS; https://www.hfpappexternal.fda.gov/scripts/fdcc/index.cfm?set=GRASNotices&id=218&sort=FDA_s_Letter&order=ASC&startrow=1&type=advanced&search=Ajinomoto) (18), making PlyP100 even more promising as a potentially safe and effective antimicrobial for the food industry. As endolysins are species- and sometimes even strain-specific, their structures and functions often differ considerably from one another (19). A deeper understanding of PlyP100 in terms of both its structure and function is important for determining optimum conditions for endolysin activity as well as for enzyme engineering efforts to make the enzyme even more efficient. Because the natural selection of bacteriophage proteins favors endolysins that bind tightly from inside the cell (8), making structurally informed modifications to PlyP100 and comparable enzymes in other *Listeria* bacteriophages could improve their function as industrially useful antimicrobials.

Herein, we sought to understand the structure and function of *listeria* phage endolysin PlyP100 using biophysical and microbiological methodology. Full-length (FL) PlyP100 was shown to be active *in vitro* and inhibited growth of *L. monocytogenes* in both standard laboratory culture conditions and a biologically relevant miniaturized laboratory queso fresco (MLQF) model. After unsuccessful attempts to obtain a crystal structure of PlyP100-FL, analysis of an AlphaFold model suggested the endolysin is actually composed of three separate domains (D1, D2 and D3) separated from one another by long flexible loop regions. To understand the structure and function of these domains, we generated PlyP100 domain truncations with different D1, D2, and D3 domain combinations that were tested in different biological conditions. From these data, we confirmed D1 to be the catalytic domain, observed that the D3 domain has cell-wall binding capacity, and determined that all three domains are required for full enzymatic function. We then solved a high-resolution crystal structure of the PlyP100 catalytic domain (D1), which was most structurally similar to endolysins from other Gram-positive bacteria. Based on this structure, we assessed the effect of eleven individual point mutations in the D1 domain, which revealed several residues that are essential for catalysis. These data provide important insights into the structure and function of PlyP100, which will aid future studies aimed towards engineering more effective versions of this food-safe antimicrobial.

## Results

### Unsuccessful crystallization of full length PlyP100

**Figure 1:**
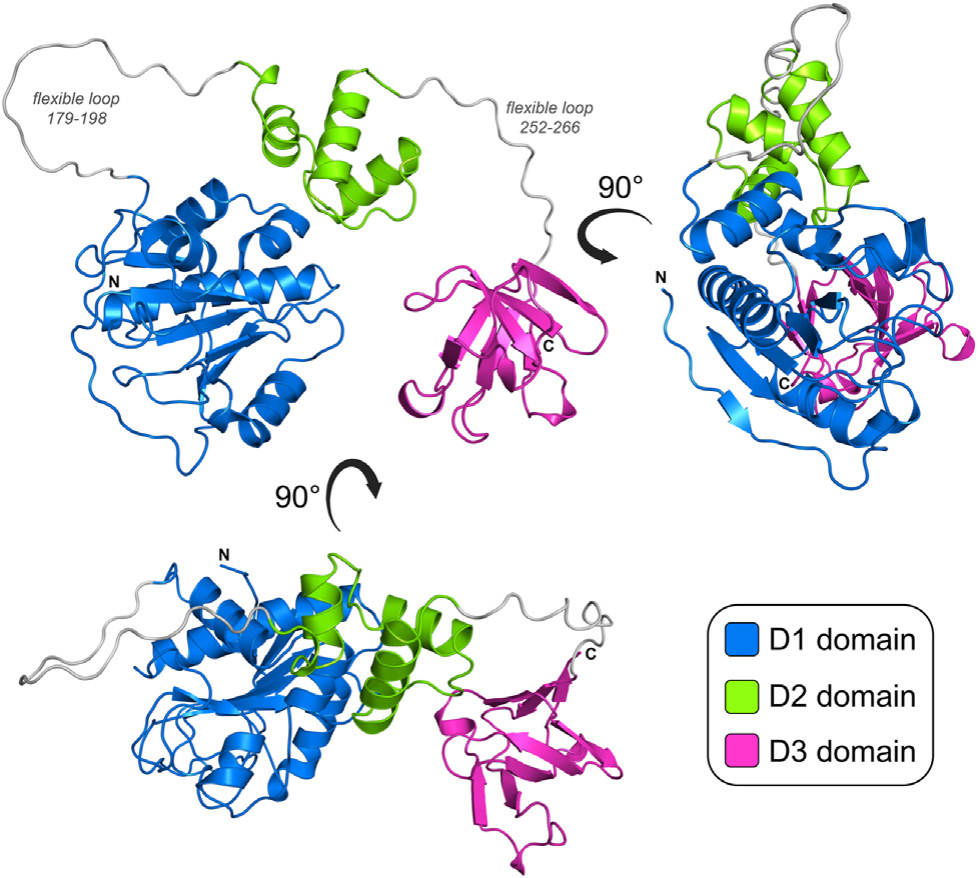
**Computational model of PlyP100.** The amino acid sequence of PlyP100-FL (UniProt ID: Q30LD5) was used to generate a predicted structure using AlphaFold (20). This analysis suggests PlyP100 contains three separate domains: D1 (*blue*, residues 1–178), D2 (*green*, residues 199–251), and D3 (*magenta*, residues 267–341). The two long flexible loop regions between these domains are colored *gray*. Figure produced with PyMOL (v.2.3.3, Schrödinger).

To gain structural insights into the PlyP100 endolysin, we sought to determine the X-ray crystal structure of the full-length enzyme (PlyP100-FL). Extensive crystallization attempts were performed, which involved testing approximately 768 crystallization conditions from eight different commercially available crystallization screens, at different protein concentrations, temperatures (room temperature and 4 °C), and different protein:precipitant ratios. Unfortunately, crystals were not obtained for PlyP100-FL in any of the conditions tested. This work was carried out prior to the development of AlphaFold (20); however, once this became available, we were able to generate a model of the full-length enzyme. This model suggested that PlyP100-FL consists of three separate domains (called D1, D2, and D3), which are separated from one another by long flexible loop regions. This includes a large N-terminal domain (D1, residues 1–178), a small intermediate domain (D2, residues 199–251), and a small C-terminal domain (D3, residues 267–341), which are structurally distinct from one another (Fig. 1). Analysis of the AlphaFold model colored according to pLDDT score indicates that while the confidence of the model prediction for the individual D1, D2, and D3 domains is “very high/confident,” the confidence of prediction for the linking regions between the domains is “low/very low” (Fig. S2). Comparison of the 5 individual models generated by AlphaFold showed that while the individual D1, D2, and D3 monomers were identical (Fig. S3*A*), the long loops linking them differed significantly (Fig. S3*B*), reflecting in their low model confidence. This supports the notion of these linker regions being flexible, which may allow for the individual D1, D2, and D3 domains to move freely relative to one another. A structural similarity search was performed for each of the individual domains using the DALI web server (21). This showed the D1 domain to be most structurally similar to the catalytic domain of amidases from other Gram-positive bacteria (Table S1), whereas the D2 and D3 domains were not found to be structurally similar to any existing proteins in the Protein Data Bank (PDB).

### Understanding the function of individual PlyP100 domains

**Figure 2:**
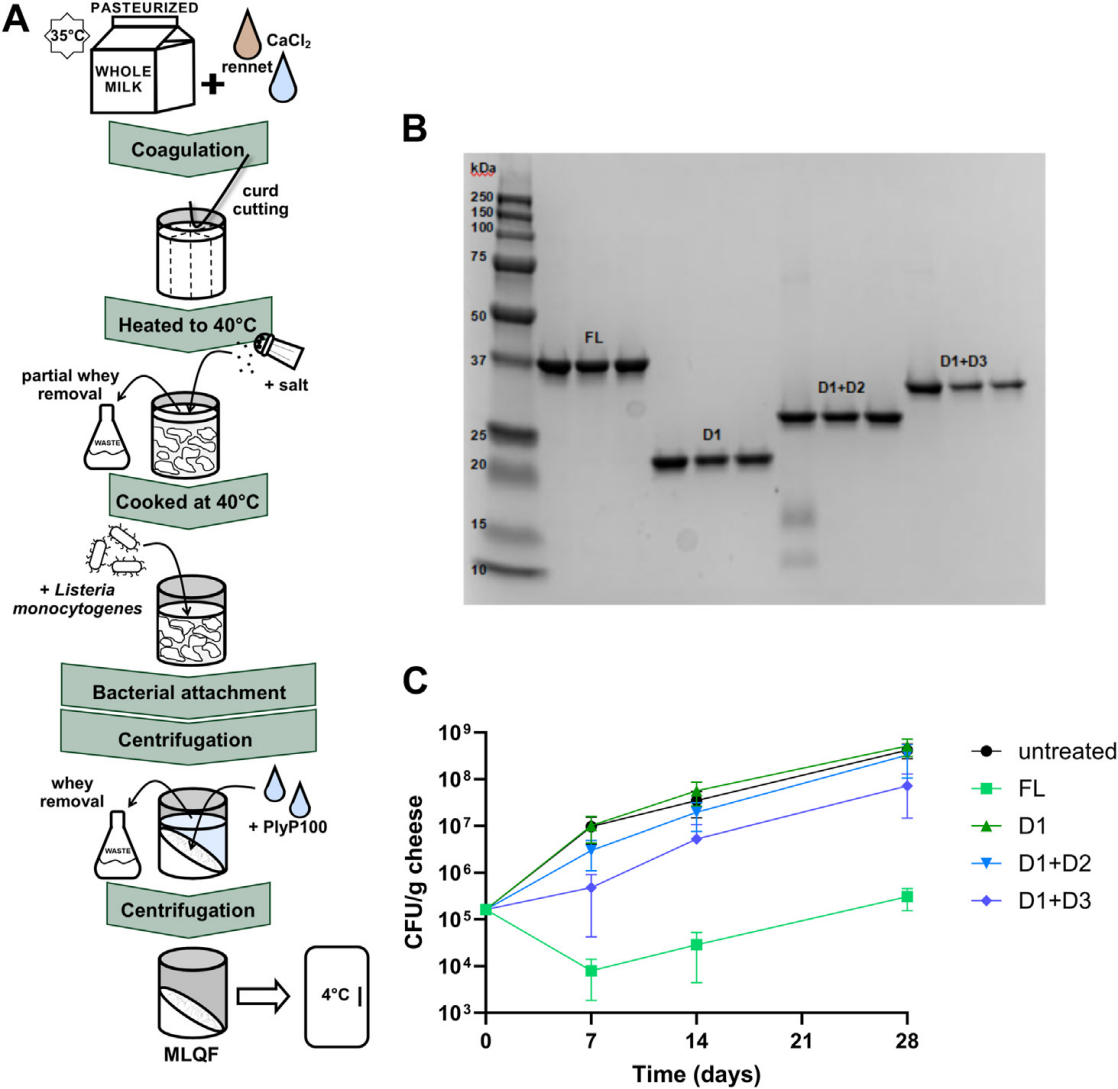
**Analysis of PlyP100 domain truncations in MLQF.***A*, schematic representation of MLQF production. *B*, SDS-PAGE analysis of purified endolysin domain truncations. Lane 1: BioRad Precision Plus Protein All Blue Standards molecular weight marker. Lanes 2 to 13: 2 μl of concentrated and buffer-exchanged purified endolysin was run after adjusting all 3 independent batches of each domain mutant endolysin to the same concentration in enzyme buffer. Domain mutant names are labeled. *C*, growth of *Listeria monocytogenes* cocktail in MLQF over 28 days. MLQF was inoculated with a cocktail of five *Listeria monocytogenes* strains and treated with 20% (v/w) 100 μM purified endolysin, for a final endolysin concentration of 0.02 μmol/g. MLQF was stored at 4 °C for 28 days. CFU/g cheese was measured on days 0, 7, 14, and 28 by diluting duplicate MLQF, plating on selective agar plates, and counting colonies after 24 h incubation. Each point is the average of six MLQF: three separate experiments each with an independent batch of endolysin in duplicate. Error bars represent standard deviation.

Based on the PlyP100-FL AlphaFold structure, which suggested the presence of three distinct domains, we produced different PlyP100 domain mutant combinations (D1 only, D1+D2, and D1+D3) for further structural and biological studies (Table S2, Fig. 2*A*). These sequences, in addition to that of PlyP100-FL, were subcloned into the expression vector pET-28a(+) so that the resulting proteins contained a C-terminal His-tag. In the case of the PlyP100-D1+D3 construct, the linker regions on either side of the middle domain were left intact so as to maintain approximately the same distance between the domains. The domain mutant endolysins were overexpressed by *E. coli* BL21(DE3) and purified by immobilized-metal affinity chromatography (Fig. 2*B*). The function of the PlyP100 domain truncations was examined using four different biological assays. Across all assays, five *Listeria monocytogenes* strains were used, chosen based on previous work (15) and pathologically relevant serotypes (Table S3). The three serotypes represented (1/2a, 1/2b, and 4b) account for almost all human infections (22). In some cases, a cocktail of these five strains was used to better represent the efficacy of PlyP100 in food-relevant settings. First, the food-relevant activity of each domain mutant was measured in a miniaturized laboratory queso fresco (MLQF) model (Fig. 2*A*) against the *L. monocytogenes* strains cocktail. Purified endolysin was added at a final concentration of 0.02 μmol/g to the curds of each MLQF and compared to sterile enzyme buffer as a vehicle control. Over a period of 28 days, PlyP100-FL had a listeriastatic effect in the MLQF at 4 °C, which is consistent with previous findings (15, 16). The domain truncations of PlyP100 (D1, D1+D2, and D1+D3) all showed decreased function against the *L. monocytogenes* cocktail over time this compared to PlyP100-FL. Twenty-eight days after inoculation with 5.2 log_10_ CFU/g *L monocytogenes*, MLQF that was treated with a buffer solution, PlyP100-D1, or PlyP100-D1+D2 contained 8.5 to 8.7 log_10_ CFU/g, while those treated with PlyP100-FL only contained 5.4 log_10_ CFU/g and those treated with PlyP100-D1+D3 contained 7.8 log_10_ CFU/g (Fig. 2*C*).

**Figure 3:**
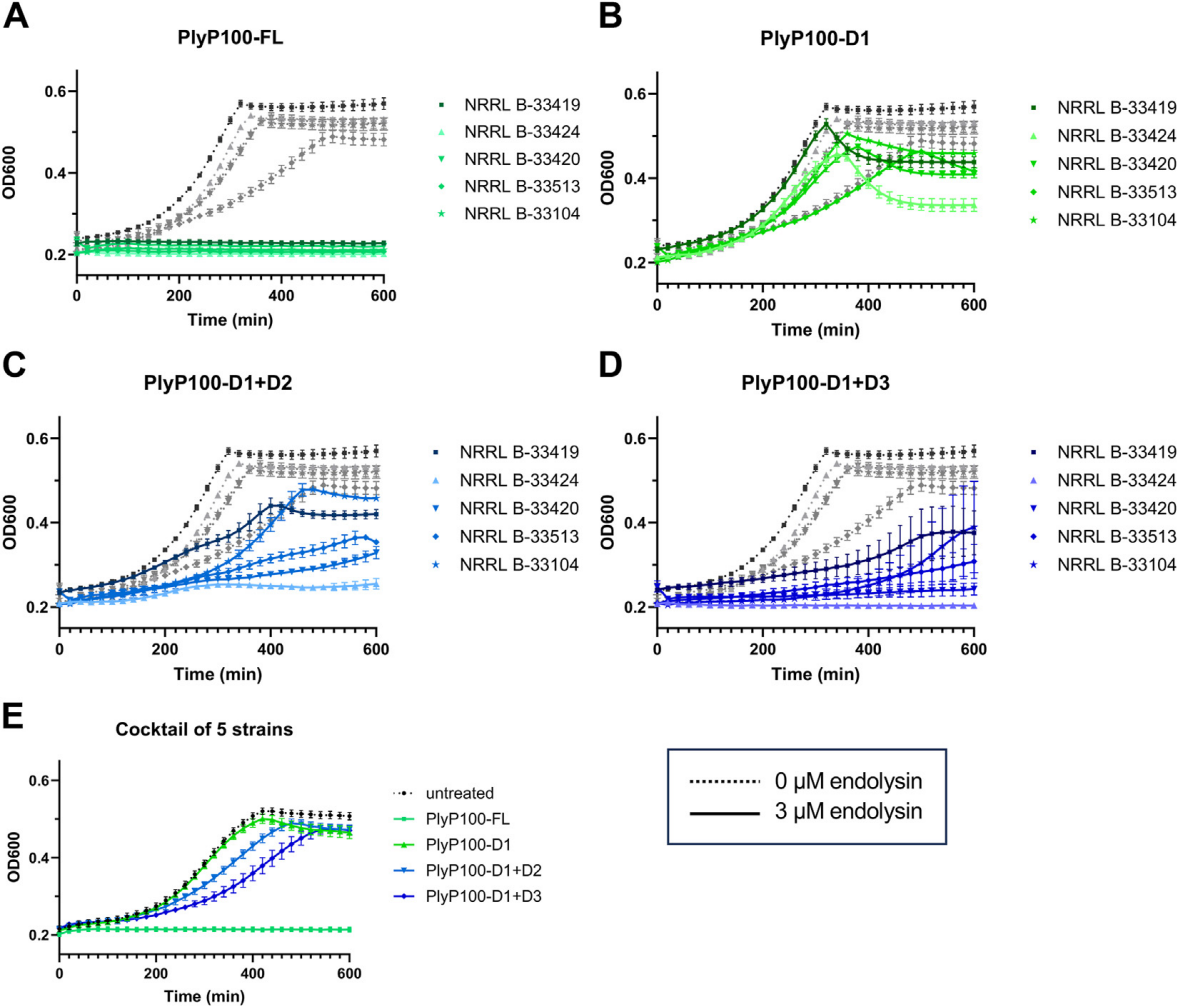
***Listeria monocytogenes* growth curves with domain mutant endolysins.** Sterile enzyme buffer with or without endolysin was added to 1% cultures of *L. monocytogenes* strains (*A*–*D*) or a 1% culture of the *L. monocytogenes* cocktail (*E*) in a 96-well plate. The cultures were incubated for 10 h at 37 °C, during which the OD_600nm_ was measured every 20 min and plotted as a curve. The untreated curves (*greyscale*, *dotted lines*) represent *L. monocytogenes* treated with enzyme buffer (50% glycerol 50% PBS), and treatment curves (*colorful, solid lines*) represent *L. monocytogenes* treated with a final concentration of 3 μM of purified endolysin in enzyme buffer. Each untreated curve is the average of 6 wells, and each treatment curve is the average of three independent batches of endolysin in triplicate (9 wells). In panels (*A*–*D*), curves represent *L. monocytogenes* strains grown independently. Dark (▪) curves are the serotype 1/2a strain, light (▲) curves are the serotype 1/2b strain, and medium (▼,◆,★) curves are serotype 4b strains. In panel (*E*), the curves represent the growth of the *L. monocytogenes* cocktail. Error bars represent standard deviation.

To further understand the functions of domains 2 and 3, the activity of each domain mutant was observed in laboratory culture conditions against each strain from the *L. monocytogenes* five-strain cocktail (Fig. 3, *A*–*D*) and the cocktail itself (Fig. 3*E*). When added at a final concentration of 3 μM to 1% cultures of *L. monocytogenes* in BHI at 37 °C, PlyP100-FL was able to completely inhibit the growth of each strain and the cocktail. Generally, PlyP100-D1+D3 showed more activity (growth inhibition) than PlyP100-D1+D2 or PlyP100-D1 alone; however, all domain truncations showed impaired activity when compared with PlyP100-FL. Interestingly, PlyP100-D1 alone did have an effect on the growth curve of all five strains, indicating that it has catalytic capacity at high cell concentrations. For all domain truncations, the *L. monocytogenes* serotype 1/2b strain was most sensitive, and the serotype 1/2a strain was least sensitive to the endolysins.

**Figure 4:**
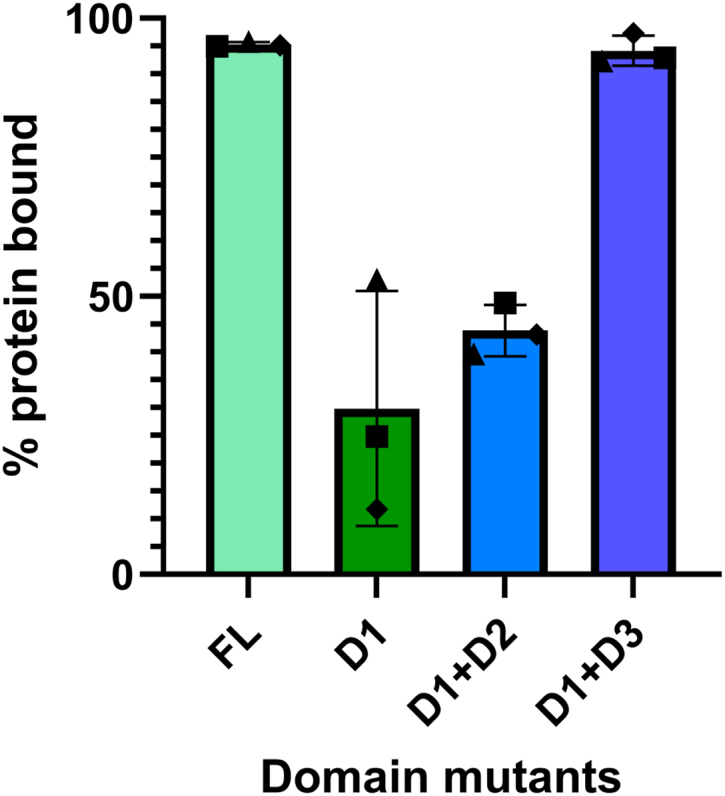
**Cell binding capacity determined by autoclaved cell assay.** Domain mutant endolysins were added at a final concentration of 0.1 μM to autoclaved *Listeria monocytogenes* cells representing each relevant serotype (NRRL B-33419, 1/2a; NRRL B-33434, 1/2b; and NRRL B-33513, 4b) suspended in PBS at a standardized OD_600nm_. After 10-min attachment at room temperature, autoclaved cell suspensions were centrifuged to pellet the insoluble cell wall debris. Endolysin remaining in supernatant (after centrifugation) was measured by His-tag ELISA and compared to the same amount (0.1 μM) of protein diluted in PBS (which represents “before” centrifugation). The “after” protein quantity in the supernatant was divided by the “before” quantity, and this value was subtracted from 100% to yield the percentage of added protein that was bound to the insoluble cell debris (and therefore not in the supernatant). Each point represents the average bound protein value of one strain of cells (1/2a ▪; 1/2b ▲; 4b⬥) measured in duplicate. Each bar represents the average of this value for all three strains of cell debris (measured in duplicate). Error bars represent the standard deviation.

The antimicrobial effect observed in PlyP100-D1 alone, in combination with the DALI search results for the PlyP100-D1 AlphaFold model, showing its structural similarity to other Gram-positive amidases, supports the role of D1 as the catalytic domain. We therefore hypothesized that domains 2 and 3 might function as cell wall-binding domains in PlyP100. To confirm this theory, PlyP100 domain mutant endolysins were incubated with autoclaved *L. monocytogenes* cells and then centrifuged to separate the insoluble cell wall fraction from the soluble fraction, after which the relative concentration of His-tagged protein remaining in the soluble fraction was measured by His-tag ELISA. These data are reported as a percentage of the initial concentration of protein that did not remain in the supernatant after centrifugation (Fig. 4). PlyP100-FL was almost entirely spun down with the cell wall debris, and PlyP100-D1+D3 showed a similar binding ability. Interestingly, when the D3 domain was absent, more than half of the protein added remained in the soluble fraction after centrifugation. This was observed even when the D2 domain was present, suggesting that D3 has a higher cell wall binding affinity than the two domains. These data support that D3 is sufficient for cell wall binding.

**Figure 5:**
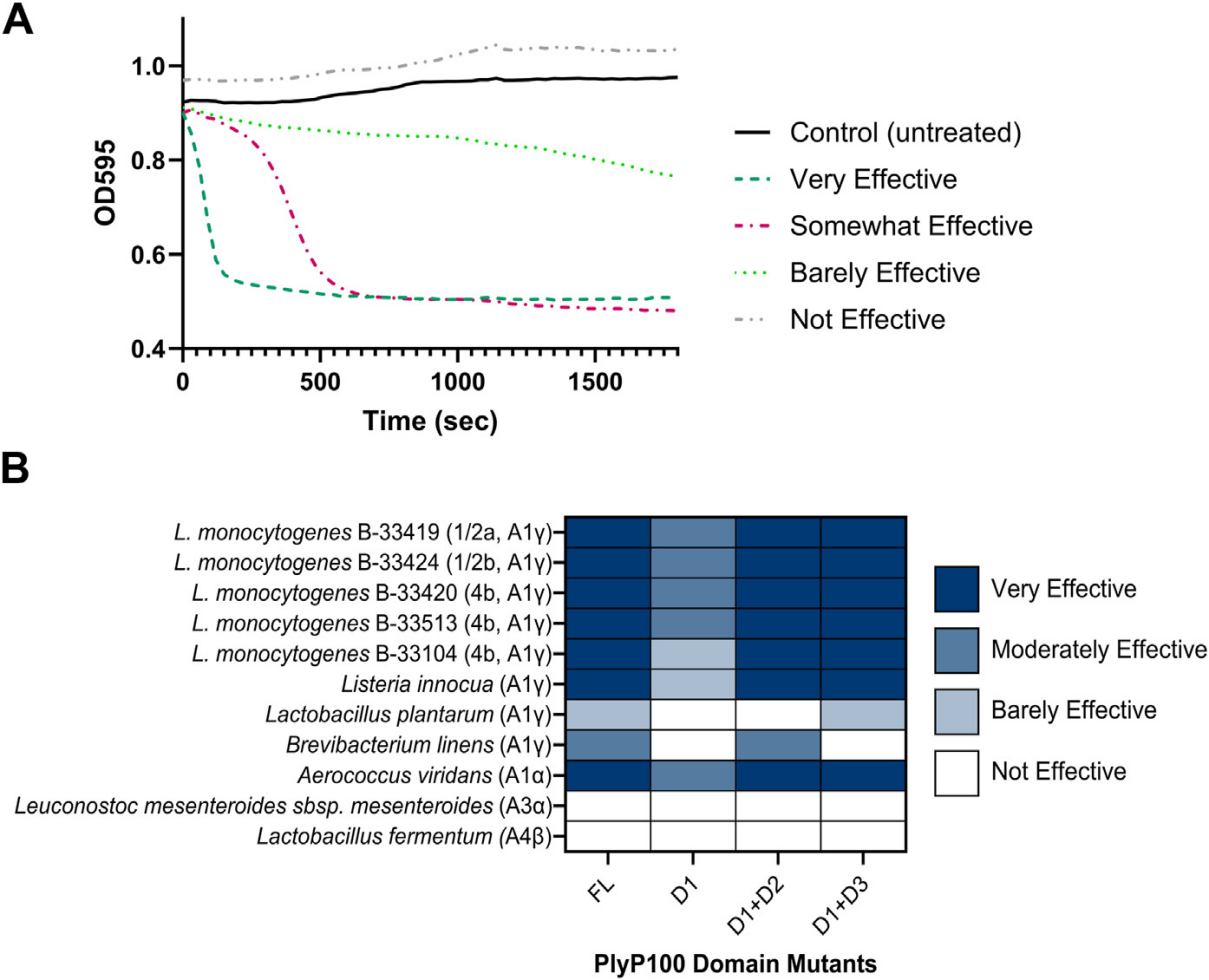
**OD reduction assay results.** Autoclaved cells of 11 Gram-positive species were tested against the endolysin domain truncations. Endolysin was added to autoclaved cells (at OD_600nm_ of 1.00 ± 0.05) at a final concentration of 1.5 μM and the OD was measured immediately and every 30 s following for 30 min. OD readings were used to plot a curve. The curves were then categorized on a 4-point scale (0 being not effective, 3 being very effective) based on the rate and extent of OD reduction. *A*, a representative curve for each category were chosen to give a visual representation of the results; Very Effective indicates a plateau in OD reduction in less than 8 min (480 s), Somewhat Effective indicates a plateau in OD reduction in more than 8 min, Barely Effective indicates OD reduction that did not plateau within 30 min, and Not Effective indicates a curve that is the same shape as the untreated control. *B*, heat maps of results for each domain mutant against each Gram-positive strain, species are listed with their serotype (for *L. monocytogenes* only) and PG type. Each combination of domain mutant and endolysin was tested in three distinct experiments, each with an independent batch of endolysin run in duplicate.

The host range of PlyP100 has previously been reported (16). To identify any domain-specific changes in this range, the domain truncations were assessed against a variety of Gram-positive species in a turbidity reduction assay. Endolysins, when active against a strain, will reduce the turbidity of a solution of cell wall debris or autoclaved cells by cutting cell wall fragments into even smaller units, making them more soluble and decreasing the optical density of the solution. The domain mutant endolysins were added to turbid resuspensions of autoclaved cells and then the optical density was measured immediately and continuously for 30 min to observe turbidity reduction. The results were then categorized rather than quantified since, in the case of PlyP100-FL and other functional endolysins, the optical density at 600 nm (OD) starts decreasing even before the plate reader can begin measuring. Results and representative curves are presented in Figure 5. PlyP100-D1 was sufficient for lysis of *L. monocytogenes* cell debris but required D2 or D3 for effective lysis. The host range of PlyP100 in this study agrees with previous research (16), with the exception that *A. viridans* was more sensitive to lysis than *Bacillus linens* or *Listeria plantarum* in the current study. Interestingly, inclusion of D2 increased efficacy against *B. linens* and not *L. plantarum,* while inclusion of D3 instead of D2 showed the opposite effect. PlyP100-FL and the domain truncations are only effective against autoclaved cells that have A1 peptidoglycan types, but enzymatic capacity is not specific to A1γ over A1α according to this and previous studies (16). This suggests that the function of the enzyme is specific to the type of interpeptide bridge found in the PG structure but not necessarily the amino acid found at position 3 of the peptide stem (23).

### Analysis of PlyP100-D1 catalytic domain—Insights into substrate binding

**Figure 6:**
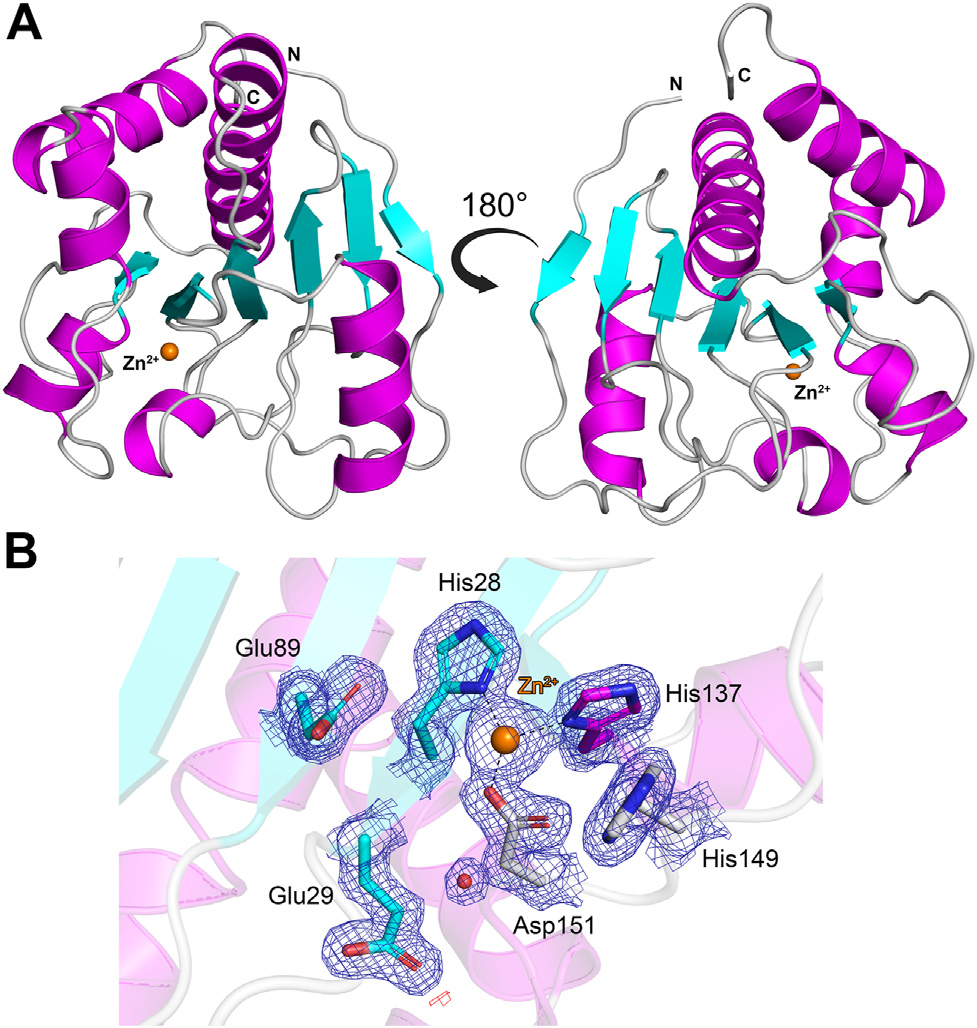
**X-ray crystal structure of the PlyP100-D1 domain.***A*, cartoon representation of the PlyP100-D1 monomer, colored according to secondary structure. Alpha-helices (α1-6) are *colored magenta*, beta-strands (β1-6) are *colored cyan*, and loop regions are *colored light grey*. The catalytic zinc ion is shown as an *orange sphere*. *B*, electron density for zinc ion binding site. The 2*F*_o_-*F*_c_ map is contoured at 1.5 σ (*blue*) and the *F*_o_-*F*_c_ maps are contoured at +3.5 σ (*green*) and −3.5 σ (*red*). Water molecules are shown as *red spheres*. Hydrogen bonds are depicted as *dashed lines*. Figure produced with PyMOL (version 3.0.4, Schrödinger).

The X-ray crystal structure of the PlyP100-D1 domain was solved to 1.80 Å resolution. The enzyme adopts a globular, mixed α/β/α fold, consisting of a central β-sheet (β1-β6) surrounded by six α-helices (α1-α6) (Fig. 6*A*). The structure contains a single zinc ion, which is coordinated by residues His28, His137, Asp151, and His177, the latter of which results from the C-terminus of a PlyP100-D1 crystal contact (Figs. 6*B*, S4*A*). The identification of this zinc was not verified experimentally; however, its assignment is supported by analysis of the site using the Check My Metal (CMM) webserver (24), and is further supported by its tetrahedral coordination, favorable hydrogen bond distances, and coordination through histidine and aspartate residues, which are consistent with what is observed for catalytic zinc binding sites in proteins (25). During refinement, it was evident that there was clear electron density for the entire C-terminal His-tag, which was non-cleavable and therefore not removed during purification (Fig. S4*B*). Thus, the interaction with His177 and the aforementioned zinc ion is not present in the native enzyme and is merely an artifact of crystallization. Superposition of PlyP100-D1 with the D1 domain of the AlphaFold model shows that while there are small differences in a number of flexible loop regions, the core structural elements superimpose well with a low RMSD of 0.48 Å (Fig. S5).

**Figure 7:**
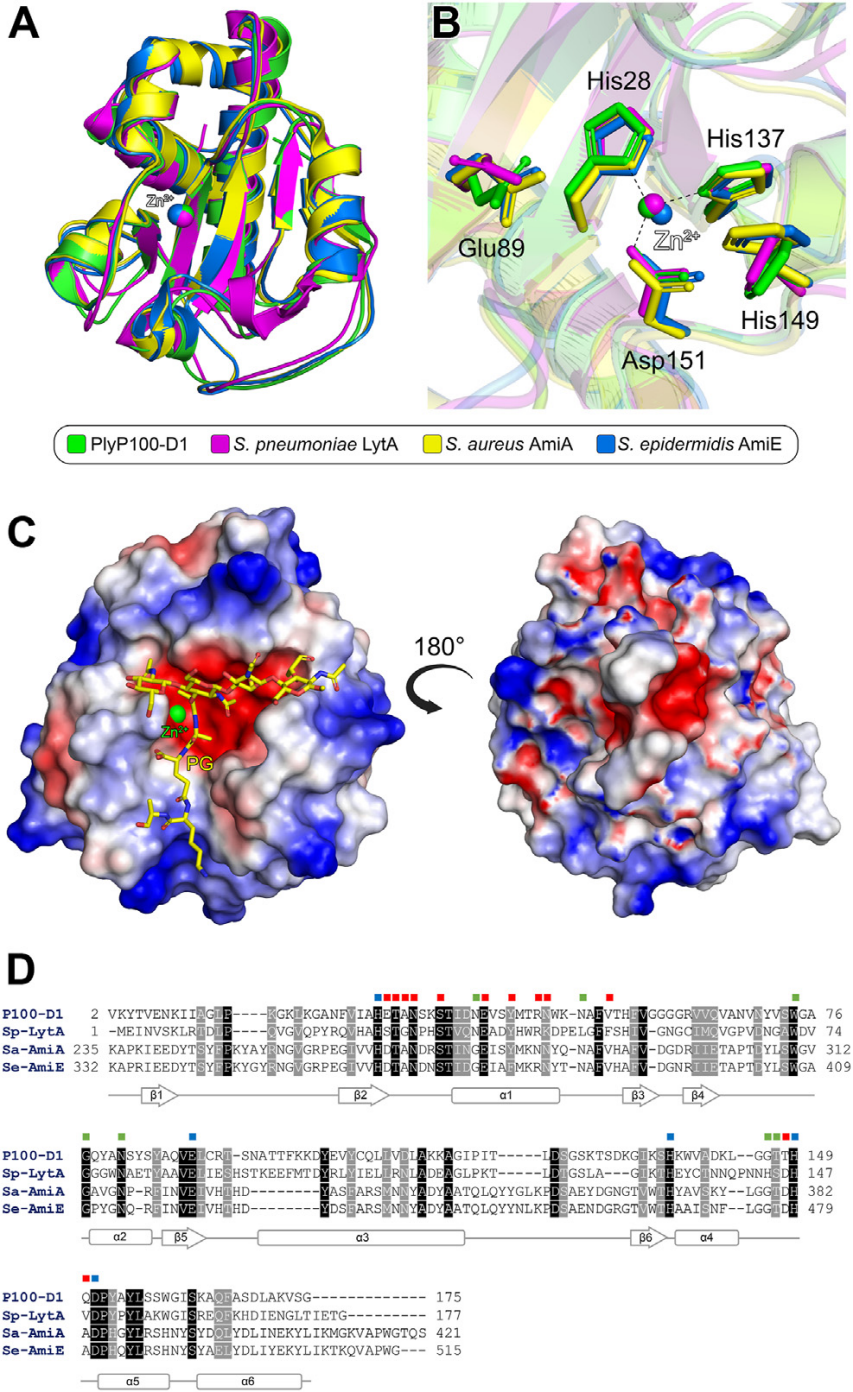
**Comparisons of PlyP100-D1 with structurally related enzymes.***A*, Cα-atom superpositions of the PlyP100-D1 (*green*) with the catalytic domains of *S. pneumoniae* LytA (*magenta*, PDB ID: 4ivv), *S. aureus* AmiA (*yellow*, PDB ID: 4knl), and *S. epidermidis* AmiE (*blue*, PDB ID: 3lat). The zinc ion from PlyP100-D1, LytA, and AmiE are shown as *green*, *magenta*, and *blue spheres*. *B*, conserved amino acids in the zinc ion binding site (PlyP100-D1 numbering). Hydrogen bonds from the PlyP100-D1 structure are depicted as *dashed lines*. *C*, superposition of PlyP100-D1 with *S. pneumoniae* LytA (PDB ID: 5ctv). Here, only the surface of PlyP100-D1 is shown. The electrostatic potential of PlyP100-D1 was calculated with APBS (64) in the range −5 kT (*red*, negative potential) to +5 kT (*blue*, positive potential). A synthetic peptidoglycan (PG) from the LytA structure is shown as a stick model; C atoms are colored *yellow*, O atoms *red*, and N atoms *blue*. The zinc atom from the PlyP100-D1 structure is shown as a *green sphere*. *D*, structure-based sequence alignment of PlyP100-D1 with LytA, AmiA, and AmiE. Identical residues are shaded *black*, *while grey* shading indicates amino acids with conserved physicochemical properties. The secondary structure annotation of PlyP100-D1 is shown below the alignment. Amino acids from PlyP100-D1 proposed to be important for peptidoglycan interaction are indicated by boxes colored *blue* (catalytic function), *red* (*glycan* interaction), or *green* (peptide interaction). Figures were produced with PyMOL (v.2.3.3, Schrödinger).

A structural similarity search was performed using the DALI webserver (21), which indicated that the PlyP100-D1 domain is most structurally similar to amidases from Gram-positive bacteria. Specifically, the enzyme displayed high Z-scores with *Streptococcus pneumoniae* LytA (26, 27, 28), *Staphylococcus aureus* AmiA (29) and *S. epidermidis* AmiE (30) (Table S4). Superposition of these amidases with PlyP100-D1 shows their core structures superimpose very well. Furthermore, the *S. pneumoniae* LytA and *S. epidermidis* AmiE structures also contain a zinc ion, which occupies the same position observed in PlyP100-D1 (Fig. 7*A*). Importantly, the residues required for zinc coordination are completely conserved between the structures, further supporting the assignment of this ion as a zinc in PlyP100-D1 (Fig. 7*B*). To gain insights into peptidoglycan (PG) substrate binding, we further compared PlyP100-D1 to a high-resolution X-ray crystal structure of *S. pneumoniae* LytA in complex with a large synthetic PG fragment (28). Following Cα-atom superposition, the PG was shown to be positioned in a mostly negatively charged Y-shaped crevice in PlyP100-D1, with the zinc ion situated at the branching point of the glycan and peptide chains of the PG substrate (Fig. 7*C*). In PlyP100-D1, the stem peptide of the PG fragment is located in the same position as the crystal contact His-tag, which hydrogen bonds with the binding pocket zinc ion (Fig. S6). Analysis of the amino acids surrounding the PG fragment in context of the current literature (28, 29, 30), suggests residues His28, Glu89, His137, His149, and Asp151 may be required for catalytic function, whereas Glu29, Thr30, Ala31, Asn32, Ser35, Glu40, Tyr43, Arg46, Asn47, Val53, Thr146, and Gln150 are likely involved in glycan binding, and residues Asn39, Ala51, Trp74, Gly77, Asn81, Gly146, and Thr147 may be important for interacting with the PG stem peptide (Fig. 7*D*). A structure-based sequence alignment of PlyP100-D1 with *S. pneumoniae* LytA, *S. aureus* AmiA, and *S. epidermidus* AmiE indicates the proposed catalytic residues are 100% conserved between the enzymes, whereas those involved in glycan and peptide binding only show 50% and 57% conservation, respectively (Fig. 7*D*). Analysis of the cell wall binding domains of LytA, AmiA, and AmiE, in the form of available X-ray crystal structures (26) or computationally generated AlphaFold models, indicates their cell wall binding domains share no structural similarity with either the D2 or the D3 domains of PlyP100 (Fig. S7).

### The effects of point mutations in the PlyP100-D1 catalytic domain

**Figure 8:**
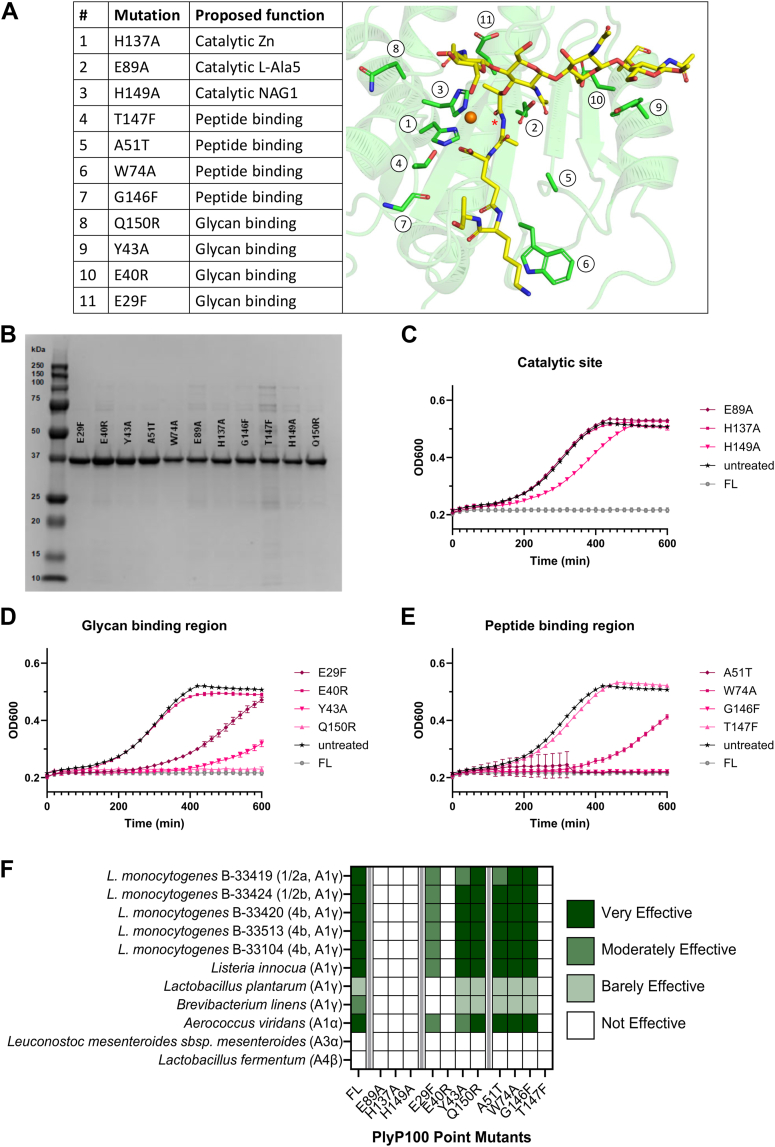
**Catalytic domain point mutants.***A*, list of the 11 different PlyP100-D1 single point mutants produced in this study. The PlyP100-D1 X-ray crystal structure (*green*) was superimposed with *S. pneumoniae* LytA (PDB ID: 5ctv) bound to an synthetic peptidoglycan fragment, which is displayed as a stick model (C atoms colored *magenta*, O atoms red and N atoms *dark blue*). The monomer of LytA is not shown for clarity. The active site zinc ion of PlyP100-D1 is shown as an *orange sphere*. The site of cleavage by PlyP100 is indicated by a *red asterisk*. The 11 listed amino acids from the proposed PlyP100-D1 active site are shown as *green sticks*. Figure produced with PyMOL (v.2.3.3, Schrödinger). *B*, SDS-PAGE analysis of purified PlyP100-FL with point mutations. Lane 1: BioRad Precision Plus Protein All Blue Standards, molecular weights are labeled next to standards. Lanes 2 to 12: equal volumes of concentrated and buffer-exchanged purified endolysin after being adjusted to the same molar concentration in enzyme buffer (50% glycerol, 50% PBS). *C–E*, *Listeria monocytogenes* growth curves. Endolysins with point mutations in the catalytic domain of PlyP100-FL were added at a final concentration of 3 μM to a 1% culture of the cocktail of strains of *L. monocytogenes* and incubated for 10 h at 37 °C in a 96-well plate. The OD_600nm_ was measured every 20 min and plotted as a curve. Each point mutant curve represents the average of three wells. Untreated curves are the average of 6 wells with the *L. monocytogenes* cocktail treated with enzyme buffer. Error bars represent standard deviation. *C*, mutations made in the proposed catalytic site, (*D*) mutations made in the proposed glycan binding region, (*E*) mutations made in the proposed peptide binding region. *F*, heat map of results of turbidity reduction assay for each domain point mutant against each Gram-positive strain, see Figure 5*A* for description of assay. Species are listed with their serotype (for *L. monocytogenes* only) and PG type. Point mutations are grouped by expected function (catalytic site E89A, H137A, H149A; glycan binding E29F, E40R, and Y43A; peptide binding A51T, W74A, G146F, T147F).

Based on our PlyP100 X-ray crystal structure and comparison to structurally similar endolysins, 11 single point mutants were generated (Fig. 8*A*). The point mutations were made within the D1 domain of PlyP100-FL. Residues were selected for their proposed significance to either the catalytic function (Glu89, His137, His149), glycan interaction (Glu29, Glu40, Tyr43, Gln150), or peptide interaction (Ala51, Trp74, Gly146, Thr147) functions of D1. Point mutant endolysins were overexpressed and purified in the same manner as the domain truncations (Fig. 8*B*). To assess whether function was retained or lost after these point mutations, the endolysins were evaluated against the *L. monocytogenes* cocktail at 37 °C in BHI broth (Fig. 8, *C*–*E*) as described earlier. PlyP100-FL can inhibit the growth of the *L. monocytogenes* cocktail for at least 10 h in these conditions, so any *L. monocytogenes* growth in this assay suggests partial loss of enzyme function.

The mutations E89A and H137A, which we propose to be important for catalytic activity, eliminated the function of PlyP100-FL against the cocktail of *L. monocytogenes* strains, and H149A severely impaired its function. Point mutations in the proposed glycan binding region of the enzyme had varied effects on its function; E29F eliminated function, E40R and Y43A impaired function to different degrees, and Q150R had no effect on the function of PlyP100-FL. Finally, some point mutations associated with the proposed peptide binding region of PlyP100-D1, namely, A51T and G146F, did not affect the function of the enzyme; however, W74A impaired function, and T147F eliminated the enzymatic activity. To determine the effects of these point mutations on the host range of PlyP100-FL, each point mutant was evaluated by a turbidity-reduction assay against each Gram-positive autoclaved cell suspension (Fig. 8*F*) in the same manner as the domain truncations were tested. The host range of the point mutation endolysins was not different from PlyP100-FL in any case. Data between the growth curve assay and turbidity reduction assay agrees that E40R, T147F, E89A, H137A, and H149A eliminate the function of PlyP100-FL, E29F impairs function substantially, A51T and Y43A impair function slightly, and Q150R, W74A, and G146F do not affect enzyme function.

### Structural comparisons with other *Listeria* phage endolysins

As our DALI search did not identify any other *Listeria* phage endolysins that are structurally similar to PlyP100-D1, we performed a general search of the PDB which indicated that X-ray crystal structures of *Listeria* phage endolysins PlyA500 (31, 32), PlyP35, PlyP40, and PlyPSA (33) have been determined (Table S5). For PlyA500 and PlyPSA, both the catalytic and cell wall binding domains have been solved, whereas for PlyP40, only the catalytic domain, and for PlyP35, only the cell wall binding domain have been solved. We therefore used AlphaFold (20) to generate computational models of these *Listeria* phage endolysins, to gain insight into the possible structures of these missing domains (Fig. S8). This suggests that PlyA500, PlyP35, and PlyPSA have a structurally similar cell-wall binding domain that is a single module, whereas PlyP40 has a cell wall binding domain consisting of two structurally distinct units. Notably, none of these cell wall binding components have any structural similarity to the AlphaFold models of the PlyP100 D2 or D3 domains (Fig. 1).

Although the DALI search did not identify any proteins that were structurally similar to the D2 and D3 domains of PlyP100, a protein BLAST (https://blast.ncbi.nlm.nih.gov/) comparison between PlyP100-FL and the whole non-redundant protein sequence database yielded nine phage proteins that have >97% sequence match and 100% query coverage, meaning the sequences are nearly identical to PlyP100. Results of the search indicate that endolysins with very similar or identical amino acid sequences can be found in *Listeria* phages LP-125, vB_LmoM_AG20, LMTA-148, LP-048, A511, LMLPA3, 20422-1, List-36, LMSP-25, and LipZ5. With this information, understanding the structure and function of PlyP100 also gives insight into these similar proteins from other *Listeria* phages, which have not yet been structurally characterized. Notably, the sequences of PlyP100 and Ply511 (from *Listeria* phage A511) only differ by a single amino acid. Unsurprisingly, the predicted AlphaFold model for Ply511 indicates the presence of three distinct domains, identical to those of PlyP100 D1, D2, and D3 (Fig. S9). This is interesting as Ply511 is one of few other *Listeria* endolysins which have been studied in some detail and expressed using other platforms (34, 35, 36, 37, 38).

## Discussion

Herein, we investigated the *L. monocytogenes* phage endolysin PlyP100 using biophysical, structural, and microbiological methodology. As the AlphaFold prediction of the PlyP100-FL structure suggested the presence of three structurally distinct domains (D1, D2, and D3) separated by long flexible loop regions, one of the main goals of this research was to identify the specific domains responsible for catalysis and cell wall binding. Our data argues that D1 is entirely responsible for catalytic activity, D3 is sufficient for cell wall binding, and D2 is necessary for full function of the enzyme, especially in food-relevant conditions.

As is common in other *Listeria* endolysins, the N-terminal domain of PlyP100, D1, is the catalytic domain (32, 33). Although PlyP100-D1 alone had little to no ability to control the growth of the *L. monocytogenes* cocktail in MLQF, and it did not prohibit the growth of any *L. monocytogenes* strain in lab culture conditions, when single point mutations in the D1 sequence were made in PlyP100-FL, enzymatic function could be eliminated. D1 alone had some enzymatic function against autoclaved cell suspensions, and the shape of the *L. monocytogenes* growth curves in lab culture conditions with D1 alone could be explained by D1 only having an effect at high cell concentrations. In the absence of any cell wall binding domains, D1 would have to come in contact with the PG by chance to cleave the substrate. It is likely that this type of interaction would still result in PG cleavage as the proposed substrate binding site of PlyP100-D1 is relatively large, shallow, and solvent-exposed. Furthermore, studies of related amidases such as *S. pneumoniae* LytA and *S. aureus* AmiA suggest a degree of structural preorganization, as their binding pockets can interact with PG substrates without having to undergo any significant structural changes (28, 29).

The clearest evidence that PlyP100-D3 is sufficient for cell wall binding comes from our simple cell binding assay. It was observed that PlyP100-FL and PlyP100-D1+D3 were equally bound to autoclaved *L. monocytogenes* cells when the His-tagged protein remaining in the supernatants of centrifuged cell suspensions was quantified by ELISA. Notably, although PlyP100-D3 is sufficient for cell wall binding in the autoclaved cell suspension, these conditions are obviously different from when *L. monocytogenes* are growing in relevant conditions, meaning PlyP100-D2 might also be useful for cell wall binding. In studies of Ply511, which AlphaFold predicts is structurally identical to PlyP100, the D2+D3 domains together have been referred to as a singular cell wall binding domain (39), which binds to the whole cell surface of *L. monocytogenes* and shows higher binding at the polar and septal regions of the cells (40). Notably, the present study is the first to identify D2 as a separate domain in PlyP100 that is not necessary for simply binding to cell walls but is important for overall enzymatic function.

Interestingly, against autoclaved cell suspensions, PlyP100-D1+D2 showed almost full enzymatic capacity compared to PlyP100-FL, which was equal to the enzymatic function observed using PlyP100-D1+D3. This means that either D2 or D3, but not both, are necessary for the lysis of autoclaved *L. monocytogenes* cells. This result, however, was not reflected in live cells in either lab or food-relevant conditions. Without D2, enzymatic function is significantly impaired compared to PlyP100-FL in both MLQF and lab culture conditions, and in both conditions, PlyP100-D1+D3 was slightly more effective than PlyP100-D1+D2. It seems that, although the domains separately have some *in vitro* functions, all three are required for function against live cells in relevant conditions, including BHI broth and MLQF. The importance of D2 and D3, in addition to the catalytic domain for inhibiting the growth of *L. monocytogenes* suggests that these domains may be important for properly targeting the PG. The flexible linker regions between the D1, D2 and D3 domains may allow them to move freely to one another, so that the cell wall binding D2 and D3 domains could act as a clamp to the PG in order to optimally position the D1 domain for effective catalysis.

Comparison of our PlyP100-D1 catalytic domain structure with *S. pneumoniae* LytA (28) bound with a peptidoglycan ligand identified the probable binding pocket of PlyP100-D1, and this allowed us to speculate on which amino acids are essential for catalytic activity. We hypothesized, based on the residues involved in PlyP100-D1 metal coordination, and their complete conservation in other structurally related amidases, that zinc binding residues His28, His137, and Asp151, in addition to the nearby residues Glu89 and His149, were likely important for catalytic function. As we predicted, mutation of His137, Glu89, and His149 to alanine completely abolished the activity of PlyP100-FL, which is in agreement with previous mutagenesis studies of the equivalent residues in *S. pneumoniae* LytA (27), *S. aureus* AmiA (29) and *S. epidermidis* AmiE (30) that show these amino acids are indispensable for enzyme activity. Altogether, this suggests that D1 of PlyP100 likely cleaves the scissile bond formed between the lactyl moiety of *N*-acetylmuramic acid and the l-Ala5 residue of the *L. monocytogenes* peptidoglycan *via* a similar mechanism to what has been observed for LytA, AmiA, and AmiE enzymes (27, 28, 29, 30). In this mechanism, an active site water molecule (not observed in our PlyP100-D1 structure) needs to be activated in order to carry out a nucleophilic attack on the carbonyl carbon of the scissile bond. In *S. aureus* AmiA and *S. epidermidis* AmiE, this water molecule coordinates the binding pocket zinc ion (alongside residues His28, His137, and Asp151; PlyP100-D1 numbering), completing its tetrahedral coordination sphere (29, 30). In those structures, the scissile bond is positioned directly adjacent to this water molecule, which is also in close proximity to nearby non-zinc binding catalytic residues Glu89 and His149 (PlyP100-D1 numbering). It has been proposed that the catalytic water becomes strongly polarized through interactions with the positively charged zinc ion and the negative carboxylate of Glu89, activating it for nucleophilic attack on the scissile bond of the substrate (27, 29, 30). This then leads to the formation of a high-energy tetrahedral reaction intermediate, which is stabilized by the protonated side chain of His149. In the next step, Glu89 acts as a proton shuttle to transfer another proton, resulting in the cleavage of the peptide bond and release of the peptide stem. Lastly, MurNAc, which is still attached to the zinc ion by the lactyl carboxyl group, is displaced by an incoming water molecule which concludes the catalytic cycle and subsequently regenerates the enzyme (28, 29, 30).

Despite the structural similarities observed between PlyP100-D1 and the catalytic domains of amidases from different bacterial species, there are marked differences in terms of the host range of these enzymes. This distinct species specificity is likely controlled on two levels: (i) recognition of the core PG structure by the catalytic domain and (ii) differences in the cell wall binding domains (CBDs) and their interactions with secondary molecules that decorate the cell surface of Gram-positive bacteria. The PG of *Listeria* belongs to the *meso-*diaminopimelic acid (*m-*DAP) cross-linked A1γ chemotype, which exhibits significant differences compared to the PG from other Gram-positive species such as *S. aureus* (41) and *S. pneumoniae* (42). Studies of *S. aureus* AmiA an *S. epidermidis* AmiE have shown that the amidase is able to efficiently hydrolyze staphylococcal PG (where the PG stem peptide consists of l-Ala-d-*i*Gln-l-Lys-d-Ala-d-Ala) but is unable to cleave *Bacillus subtilis* PG (l-Ala-d-*i*Glu-*m-*DAP-d-Ala-d-Ala) (29, 43), where the stem peptide is more similar to what is observed in *L. monocytogenes* (13). Interestingly, substitutions in the peptide stem of either d-*i*Gln to d-*i*Glu or l-Lys to *m-*DAP on their own seemed to be tolerated, as AmiE was still able to cleave the two substrates, albeit with lower efficiency (44). The inability of Staphylococcal endolysins to hydrolyze *B. subtilis* PG was therefore proposed to be due to a cumulative effect of these differences in the peptide stem, but also due to differences in cross-linking between peptide stems in the PG (29). Specifically, in *S. aureus* and *S. epidermidis,* l-lysine (at position 3) of one stem is linked *via* a pentaglycine bridge to d-alanine (at position 4) of the second peptide stem (41), whereas in *B. subtilis* (and also *L. monocytogenes*) *meso*-DAP (at position 3) is directly cross-linked to the d-alanine (at position 4) in the second peptide (13, 43). Overall, these two factors influence the optimal positioning of the PG substrate in the binding pocket of the catalytic domain. This agrees with our PlyP100 mutagenesis studies, which identified non-catalytic glycan and peptide-interacting residues (Glu40 and Thr147), which, when mutated, completely inactivated the enzyme. As these residues were changed to amino acids that were larger and/or had the opposite charge, it is evident that their effects on enzyme activity result from structural perturbations in substrate binding, highlighting the importance of stringent substrate positioning for effective PG hydrolysis.

It has been observed and generally accepted that endolysins have a modular structure and that domains can be swapped to change specificity (45). Extensive studies have also shown that cell wall teichoic acids (TAs) and lipoteichoic acids (LTAs) are important for the anchoring and specificity of the CBDs of *S. pneumoniae* LytA (26, 46, 47, 48) and *S. epiderimidis* AmiE (49, 50) towards the Streptococcal and Staphylococcal cell wall, respectively. The CBDs are also proposed to be responsible for the host ranges of *Listeria* phage endolysins: Ply118 primarily binds serotype half strains, PlyA500 and PlyPSA bind serotype 4, 5, and 6 strains, PlyP35, Ply511, and PlyP40 bind a wider range of strains, and the host ranges for these CBDs are consistent with the host ranges of their associated phages (40). In the present study, we noted a marked difference in efficacy between strains of *L. monocytogenes* representing different serotypes. The serotype 1/2b strain was most sensitive and the serotype 1/2a strain was the least sensitive to PlyP100-FL and all domain truncations, and the sensitivities of serotype 4b strains were variable but generally fell somewhere in the middle. As the PG structure between these strains is conserved, these differences are mostly attributed to other components of the cell surface. First, serotypes are determined by O- (somatic) and H- (flagellar) antigens, which describe the surface and flagella of the cells. Serotypes 1/2a and 1/2b share O-antigens but have slightly different H-antigen combinations, and serotype 4b has the same combination of H-antigens as serotype 1/2b, but entirely different O-antigens than either 1/2a or 1/2b (51). Second, the wall teichoic acids (WTA) that are linked to the glycan strands of PG in the cell wall are known to be different across *L. monocytogenes* serotypes (52). WTAs are carbohydrates that make up a major component of the cell wall and can have a variety of structures. WTAs have previously been cited to restrict access to PG for *Listeria* endolysin CBDs, including the CBD of Ply511, which is synonymous with D2 and D3 of PlyP100 (39). Notably, the WTAs in 1/2a and 1/2b strains (composed of rhamnose, glucosamine, and phosphorus) are known to have a different structure than WTAs found in 4b strains (composed of glucose, galactose, and glucosamine) (52). These WTAs might also be glycosylated differently across serotypes (53, 54). Finally, it is important to note that, although serotypes 1/2a and 1/2b share similar WTAs, they have been determined to come from distinct evolutionary lineages based on an evaluation of other antigenic genes (55).

The differences in these cell surface proteins, flagellar proteins, and cell wall carbohydrates may affect the ability of PlyP100 to reach the PG layer. We speculate that this could be due to decreased cell wall binding capacity by D2 and D3, as the CBD of other *Listeria* endolysins has previously been cited as the reason for endolysin serotype specificity (56). However, differences were also observed when D1 alone was used against each *L. monocytogenes* strain, so the catalytic activity itself or the steric hindrance of even small proteins might be affected by these serotype variations. These differences in cell surface proteins and carbohydrates could also explain why PlyP100-D1+D2 showed activity against *B. linens* and not *L. plantarum,* while PlyP100-D1+D3 showed the opposite effect. The cell surfaces of *B. linens* and *L. plantarum* may be accessible to differently sized and/or shaped molecules.

Overall, the functional and structural insights presented here will be highly valuable for the future development of more effective versions of PlyP100 and/or identical *Listeria* phage endolysins, for use as novel food-safe antimicrobials. Furthermore, understanding each domain separately will contribute to the base of knowledge for developing modular endolysins to improve food safety.

## Materials and methods

### Cell culture and growth conditions

All strains used in this work are listed in Table S3. Cells were grown in accordance with American Type Culture Collection (ATCC) growth condition recommendations (https://www.atcc.org/). *Listeria* strains were grown in Brain Heart Infusion (BHI) broth (Millipore-Sigma), lactic acid bacteria were grown in DeMan, Rogosa, and Sharpe (MRS, Hardy Diagnostics), and *B. linens* was grown in Tryptic Soy Broth (TSB, Hardy Diagnostics). The 5 L. *monocytogenes* strains used were chosen based on previous work and are biologically relevant since they were isolated from foods or food-borne epidemics (15). Other Gram-positive strains were chosen based on their peptidoglycan chemotype and/or relevance to the food industry.

### Plasmid construction and cloning

Plasmids were synthesized by Twist Bioscience. The endolysin PlyP100 sequence was identified in the *Listeria* phage P100 genome sequence (GenBank: DQ004855.1) and cloned into pET-28a(+) using restriction enzymes NcoI and XhoI so that the resulting protein contained a C-terminal His-tag. The protein sequences for all synthesized domain truncations are listed in Table S2. To achieve individual point mutations, codons were switched in the FL PlyP100 sequence to the highest frequency codon for a selected residue in *E. coli* (https://www.genscript.com/tools/codon-frequency-table). Synthesized plasmids were then transformed into *E. coli* BL21(DE3) by heat shock at 42 °C and plated onto LB agar supplemented with 50 μg/ml kanamycin (Fisher BioReagents). Glycerol stocks were made from a single transformant colony and stored at −80 °C until use.

### Protein purification for biological assays

Following production of overnight *E. coli* BL21(DE3) pre-cultures, cells were grown in LB broth containing 50 μg/μl kanamycin at 37 °C with constant shaking, until mid-log phase (OD approximately 0.4–0.5). The cells were then induced with 0.8 mM IPTG and incubated at 15 °C overnight with shaking at 150 rpm. The following day, the cells were harvested by centrifugation and resuspended in lysis buffer (50 mM NaH_2_PO_4_, 300 mM NaCl, 10 mM imidazole, pH 8.0) supplemented with 1 mM PMSF and 2 mg/ml lysozyme. Resuspended cells were incubated on ice for 1 hour before sonication, after which the lysate was clarified by centrifugation. The soluble supernatant was filtered with a 0.22 μM syringe filter and added to Ni-NTA Agarose beads (Invitrogen). After 1 h incubation at 4 °C with rotation, the protein-bead slurry was poured onto a Poly-Prep Chromatography Column (Bio-Rad). The beads were washed with 10 CV of wash buffer (50 mM NaH_2_PO_4_, 300 mM NaCl, 20 mM imidazole, pH 8.0), after which bound protein was eluted with 5 CV of elution buffer (50 mM NaH_2_PO_4_, 300 mM NaCl, 250 mM imidazole, pH 8.0). The elution fraction was then concentrated and buffer exchanged into PBS (Cytiva) using an Amicon Ultra 10 kDa MWCO centrifugal filter (Millipore-Sigma). The protein was then added to an equal volume of glycerol and sterile filtered before storage at −20 °C. Protein purity was verified by SDS-PAGE. Independent batches of endolysins were started from the same glycerol stock of transformed *E. coli* BL21(DE3) but grown, induced, harvested, and purified independently on different days.

### OD reduction assay

Gram-positive strains were recovered from glycerol stocks stored at −80 °C and subcultured in appropriate growth conditions. Cultures (100 ml) of each strain were grown to stationary phase, after which the cells were pelleted and resuspended in PBS buffer. Washed cells were autoclaved for 20 min at 121 °C, left to cool, and then adjusted to OD of 1.00 ± 0.05 using PBS buffer. The autoclaved cell suspensions were stored at 4 °C for up to 1 week before use or were pelleted and stored at −80 °C for later use. A 10 μl volume of enzyme buffer (50% glycerol, 50% PBS) with each purified endolysin (15 μM) was aliquoted into a 96-well plate at room temperature. Enzyme buffer without endolysin was used as a negative control. Using a multichannel pipette, 90 μl of autoclaved cell suspension was added to each well, after which the OD was immediately measured using a Multiskan Ascent plate reader (Thermo Electron Corporation). The OD was then read every 30 s for 30 min, with a 2-s orbital shake before each read. OD reduction curves were then qualitatively analyzed and compared with a control curve.

### Growth curve assay

*Listeria* strains were recovered from glycerol stocks stored at −80 °C and grown to stationary phase overnight at 37 °C with constant shaking at 200 rpm. Overnight culture was passed into sterile BHI broth, to make a 1% culture, immediately before the growth curve assay; for individual strains, 200 μl stationary phase culture was added to 19.8 ml BHI, for the cocktail, 40 μl of each of five *Listeria monocytogenes* strains was added to 19.8 ml BHI. In a 96-well plate, 20 μl of sterile enzyme buffer (50% glycerol, 50% PBS) with or without purified endolysin (30 μM) was added to 180 μl of the 1% culture. The plate was then covered with a polyurethane sealing membrane (MilliporeSigma) and incubated in a FilterMax F5 microplate reader (Molecular Devices) for 10 h at 37 °C. The OD was read every 20 min, with a 5-s orbital shake before each read. The growth curve for each strain/endolysin mutant combination was measured in triplicate.

### Miniaturized laboratory queso fresco (MLQF) assay

Each of the five cocktail strains of *L. monocytogenes* cultures were started from −80 °C glycerol stocks and grown to ∼9 log CFU/ml overnight at 37 °C with constant shaking at 200 rpm. A 20 μl volume of each cocktail strain was added to 9.9 ml PBS, vortexed, and then diluted further (100 μl in 9.9 ml PBS) to yield a ∼5 log CFU/ml dilution of the *L. monocytogenes* cocktail. This dilution was stored at 4 °C for no more than 4 h before application to MLQF. MLQF was prepared as previously described (57) with slight adjustments. A 50 ml volume of pasteurized whole milk was warmed to 35 °C, after which 50 μl of commercial rennet (North Mountain Supply) diluted in 50 μl deionized (DI) water and 20 μl of 4.5 M CaCl_2_ diluted in 180 uL DI water were added to the milk. The milk solution was distributed as 1 ml aliquots in o-ring lidded 2 ml microcentrifuge tubes. The tubes were incubated at 35 °C for 45 min in a heat block, following which the curds were cut with a bent inoculating needle before being returned to the heat block and incubated for a further 30 min. During this incubation step, the temperature increased by 1 °C every 6 min until the temperature reached 40 °C. The tubes were then centrifuged at 1000 g for 30 s to 1 min, after which 200 μl of whey was removed and discarded from each tube. A 50 μl volume of 2.7 M NaCl was then added and stirred into the curds. The tubes were then warmed again for 20 min at 40 °C. The ∼5 log CFU/ml dilution of *Listeria monocytogenes* cocktail was then added to the curds and whey in a 1:1 (v/w) ratio, according to the average MLQF final weight (approximately 220 mg) and left at room temperature for 15 min to allow for bacterial attachment. The tubes were centrifuged at 6010*g* for 5 min at 4 °C, following which the whey was poured off. To the dry curds, 44 μl of purified endolysin (100 μM) or sterile enzyme buffer (50% glycerol, 50% PBS) was added for a final concentration of 0.02 μmol endolysin per gram MLQF (0.02 μmol/g), and thoroughly stirred using a pipette tip. The tubes were centrifuged again at 6010*g* for 8 min at 4 °C and the remaining whey was then removed with a micropipette. MLQF were stored at 4 °C for up to 28 days. *L. monocytogenes* growth was quantified by enumeration on days 0, 7, 14, and 28 of storage. On the day the cheese was made (Day 0), 2 untreated MLQF were enumerated, and on days 7, 14, and 28, duplicate MLQF from each treatment group were enumerated. For enumeration, 1.96 ml sterile PBS was added to a MLQF and mixed thoroughly by pipetting and vortexing, and then serially diluted in PBS. Appropriate dilutions were plated in duplicate using an Eddy Jet spiral plater (IUL instruments) on agar plates made from PALCAM base (Neogen) supplemented with 20 μg/ml Ceftazidime. The spiral plater was set to E mode (50 μl) and plates were counted using a Flash & Go colony counter (IUL) after 24 to 36 h incubation at 37 °C.

### His-tag ELISA for identification of cell wall binding domain

Domain mutant endolysins were added at a final concentration of 0.1 μM to autoclaved *Listeria monocytogenes* cells representing each relevant serotype (NRRL B-33419 1/2a, NRRL B-33424 1/2b, and NRRL B-33513 4b) suspended in PBS at an OD of 1 ± 0.05 (see turbidity reduction assay method for more details on autoclaved cell suspensions). After 10-minute attachment at room temperature, autoclaved cell suspensions were centrifuged at 16,000 g for 5 min to pellet the insoluble cell wall debris. Endolysin remaining in the supernatant (“after” centrifugation) was measured by His-tag ELISA (GenScript cat. no. L00436) and compared to the same amount (0.1 μM) of protein diluted in PBS (which represents “before” centrifugation). The “after” protein concentration in the supernatant was divided by the “before” concentration, and this value was subtracted from 100% to yield the percent of added protein that was bound to the insoluble cell debris (and therefore not in the supernatant). Each of the three serotypes of autoclaved cells was tested in duplicate.

### Expression and purification of PlyP100 catalytic domain for X-ray crystallography

PlyP100-D1 DNA optimized for *E. coli* expression was purchased from GenScript and subcloned into pET24a(+) (Novagen). His-tagged PlyP100-D1 (Table S2) was expressed in *E. coli* BL21 cells overnight at 18 °C using a LEX Bioreactor. The cells were harvested and resuspended in lysis buffer (50 mM HEPES pH 8.0, 500 mM NaCl, 10% glycerol, 0.5 mM TCEP, 10 mM imidazole, 25 U/ml benzonase, 1 μl/ml Roche protease cocktail inhibitor) and then lysed *via* sonication. The lysate was clarified by ultracentrifugation for 1 h at 42,000 rpm. PlyP100-D1 was purified to homogeneity using Immobilized metal affinity chromatography (IMAC) followed by Size-exclusion chromatography using a Superdex 200 16/600 column. The final protein storage buffer contained 20 mM HEPES pH 7.5, 300 mM NaCl, 10% glycerol and 0.5 mM TCEP. Protein aliquots were flash frozen in liquid nitrogen, and stored at −80 °C. The protein was verified to be >95% pure using SDS-PAGE.

### Crystallization of PlyP100 catalytic domain

An aliquot of purified PlyP100 catalytic domain (45 mg/ml) was preincubated with 10 mM *N*-acetylmuramic acid. The protein sample was crystallized *via* sitting drop vapor diffusion in 0.2 M sodium sulfate, 20% (v/v) PEG3350 at 4 °C. Protein crystals that appeared after 2 weeks were soaked briefly in a cryo-protectant solution consisting of the respective growth condition supplemented with 20% (v/v) glycerol, before flash freezing in liquid nitrogen.

### Data collection, structure determination, and refinement

X-ray diffraction data were collected on the I03 beamline of the Diamond Light Source. A dataset was collected at 100 K using a single crystal. The resulting data were indexed and integrated with DIALS (58) and scaled using AIMLESS (59) within the CCP4 suite (60). The structure was solved *via* molecular replacement with PHASER (61) using the monomer of *S. pneumoniae* LytA (PDB ID: 5CTV) as the search model. Several rounds of manual model building and refinement were performed using Coot (62) and REFMAC5 (63) during which waters and ligands were incorporated into the structure. Data processing and refinement statistics are presented in Table S6. The coordinates and structure factors for this structure were deposited in the PDB under the accession code 9HTU.

## Data availability

The protein structure presented in this paper has been deposited in the Protein Data Bank (PDB) under the accession code 9HTU. All remaining data are contained within the article.

## Supporting information

This article contains supporting information (20, 26, 27, 28, 29, 30, 31, 32, 33, 65, 66, 67, 68).

## Conflict of interest

The authors declare that they have no conflicts of interest with the contents of this article.
