# Supplementary material for: Structural and functional insights into Listeriamonocytogenes phage endolysin PlyP100: A promising food safety tool
Source: J Biol Chem. 2025 May 27;301(7):110295. doi: 10.1016/j.jbc.2025.110295 (PMC12221725; doi:10.1016/j.jbc.2025.110295)
Supplement: Supporting information [file mmc1.docx]

**Structural and functional insights into *Listeria monocytogenes* phage endolysin PlyP100 – a promising food safety tool**

Karson R. Bateman^1†^, Emma Scaletti Hutchinson^2†^, Göran Widmalm^3^, Michael J. Miller^1*^ and Pål Stenmark^2*^

^1^Food Science and Human Nutrition, University of Illinois Urbana-Champaign, Urbana, IL 81801, USA

^2^Department of Biochemistry and Biophysics, Stockholm University, SE-106 91 Stockholm, Sweden

^3^Department of Chemistry, Stockholm University, SE-106 91 Stockholm, Sweden

^†^Authors contributed equally to this work

^*^Correspondence and requests for materials should be addressed to Prof. Michael J. Miller ([mille216@illinois.edu](mailto:mille216@illinois.edu)) or Prof. Pål Stenmark ([stenmark@dbb.su.se](mailto:stenmark@dbb.su.se))


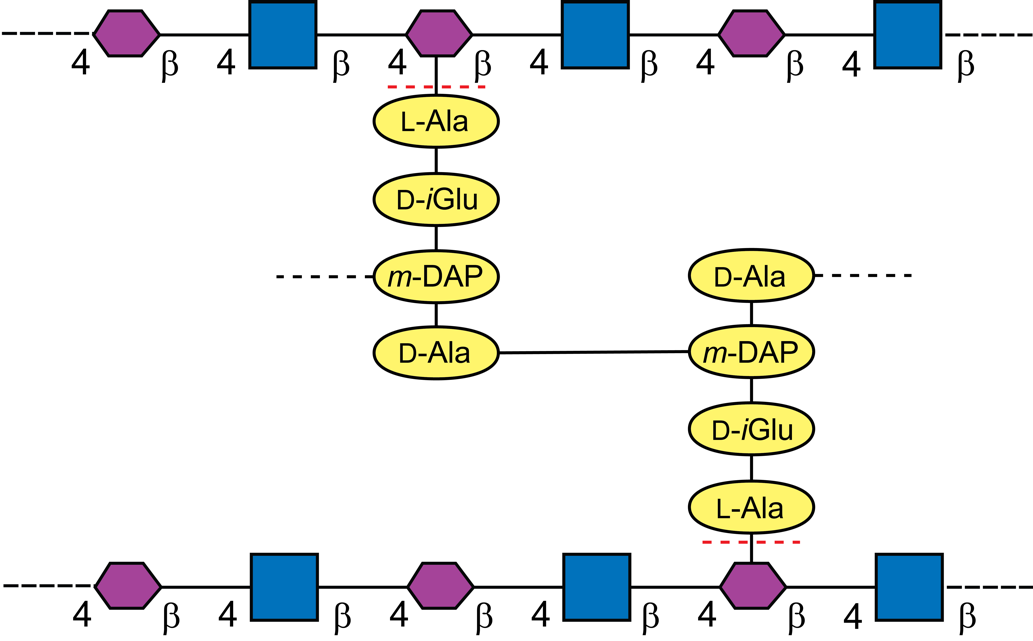


**Figure S1. Schematic of peptidoglycan structure from *L. monocytogenes*.** The polysaccharide component consists of alternating d-GlcNAc (blue square) and MurNAc (purple hexagon) residues, drawn in SNFG format (1, 2) both of which are β-(1→4)-linked (3). l-Alanine residues are joined to the lactyl group of MurNAc residues by an amide linkage and the peptides are directly crosslinked between d-Ala in one chain and *m*-DAP in another chain, thereby forming a mesh‑like network together with the polysaccharide constituent; d-*i*Glu = d‑*iso*‑glutamic acid, *m*‑DAP = *meso*‑2,6‑diaminopimelic acid. The sites of cleavage by the amidase Ply100 are indicated by red dashed lines.

**
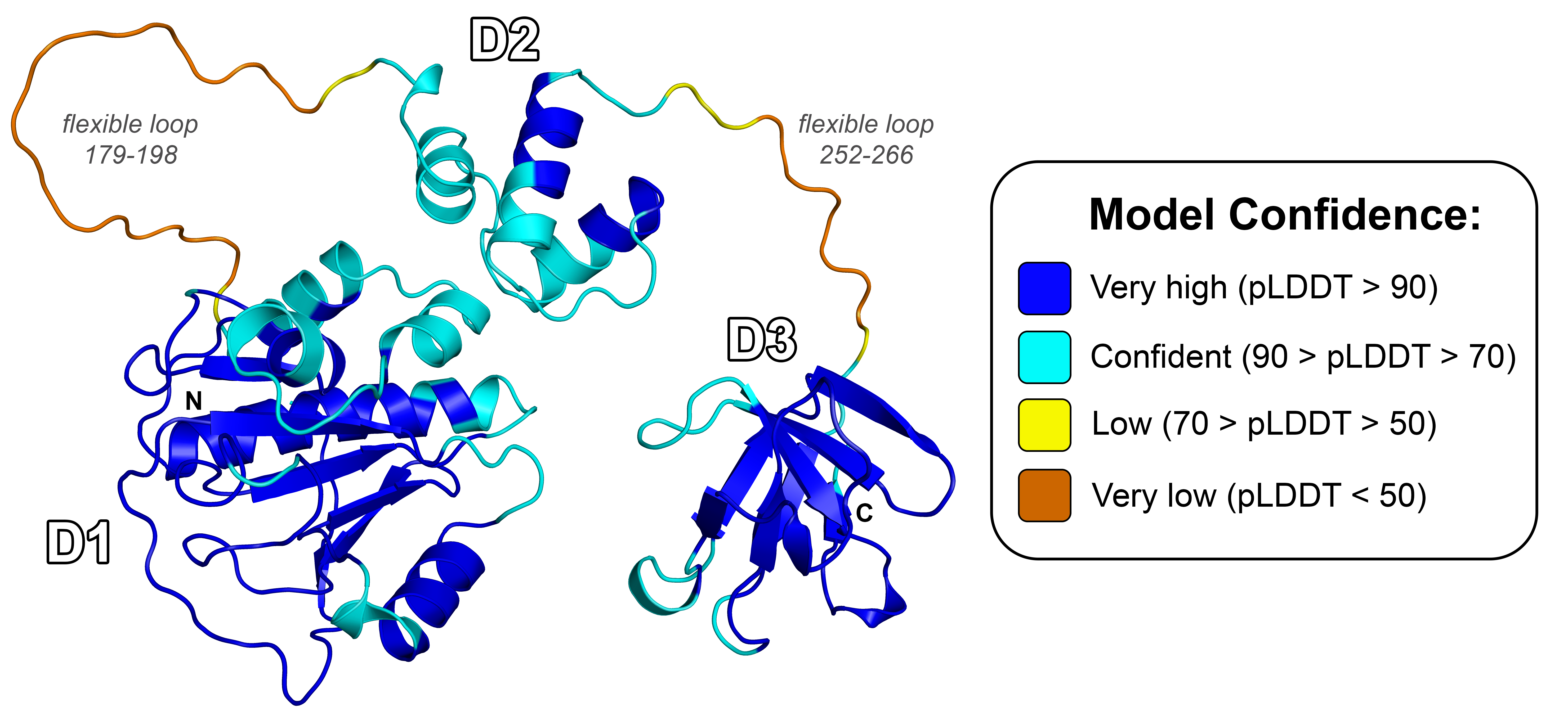
**

**Figure S2. Confidence of PlyP100 computational model.** The amino acid sequence of PlyP100-FL (UniProt ID: Q30LD5) was used to generate a predicted structure using AlphaFold (4). PlyP100-FL contains three separate domains: D1 (residues 1-178), D2 (residues 199-251), and D3 (residues 267-341). The two long flexible loop regions between these domains are indicated. The cartoon is colored according to pLDDT score, which indicates the quality of the PlyP100 model. Figure produced with PyMOL (v.2.3.3, Schrödinger).

**
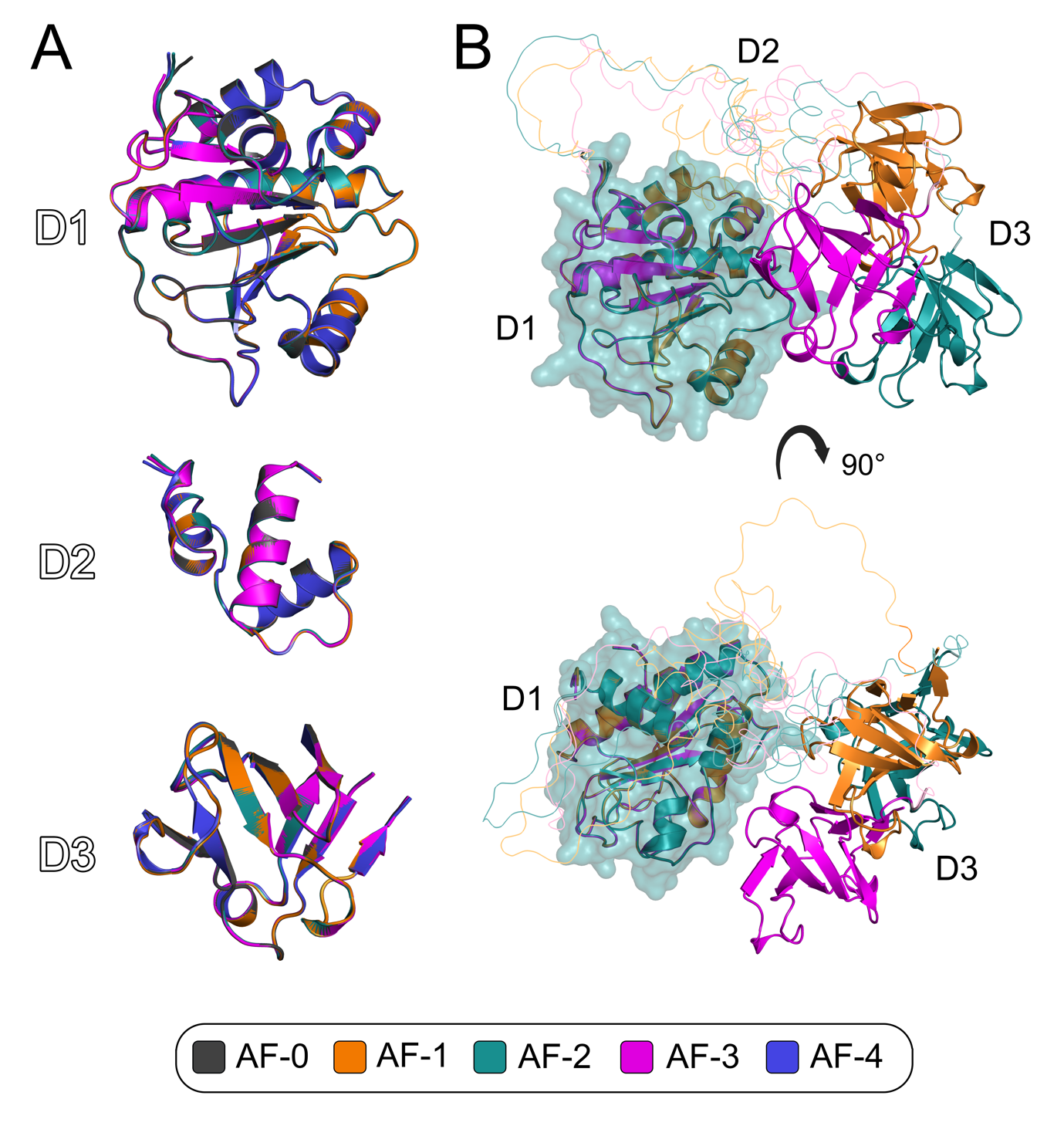
**

**Figure S3. Comparison of individual PlyP100-D1 models generated using AlphaFold.** The amino acid sequence of PlyP100-FL (UniProt ID: Q30LD5) was used to produce a predicted structure using AlphaFold (4). This resulted in five PlyP100-FL models: AF-0 (dark grey), AF-1 (orange), AF-2 (dark teal), AF-3 (magenta) and AF-4 (blue). **(A)** Cα-atom superposition of the separate D1, D2 and D3 domains for the individual AlphaFold models. **(B)** Comparison of full-length structural models using AF-2, AF-3 and AF-4 as representative examples, where only the D1 domain from the models are superimposed. This highlights the significant differences with the flexible loop regions of the models. The D1 domain from model AF-2 is shown as a semi-transparent surface. In this panel, the D2 domains and the flexible loop regions linking the domains are displayed as thin ribbons for clarity. Figure produced with PyMOL (version 3.0.4, Schrödinger).

**
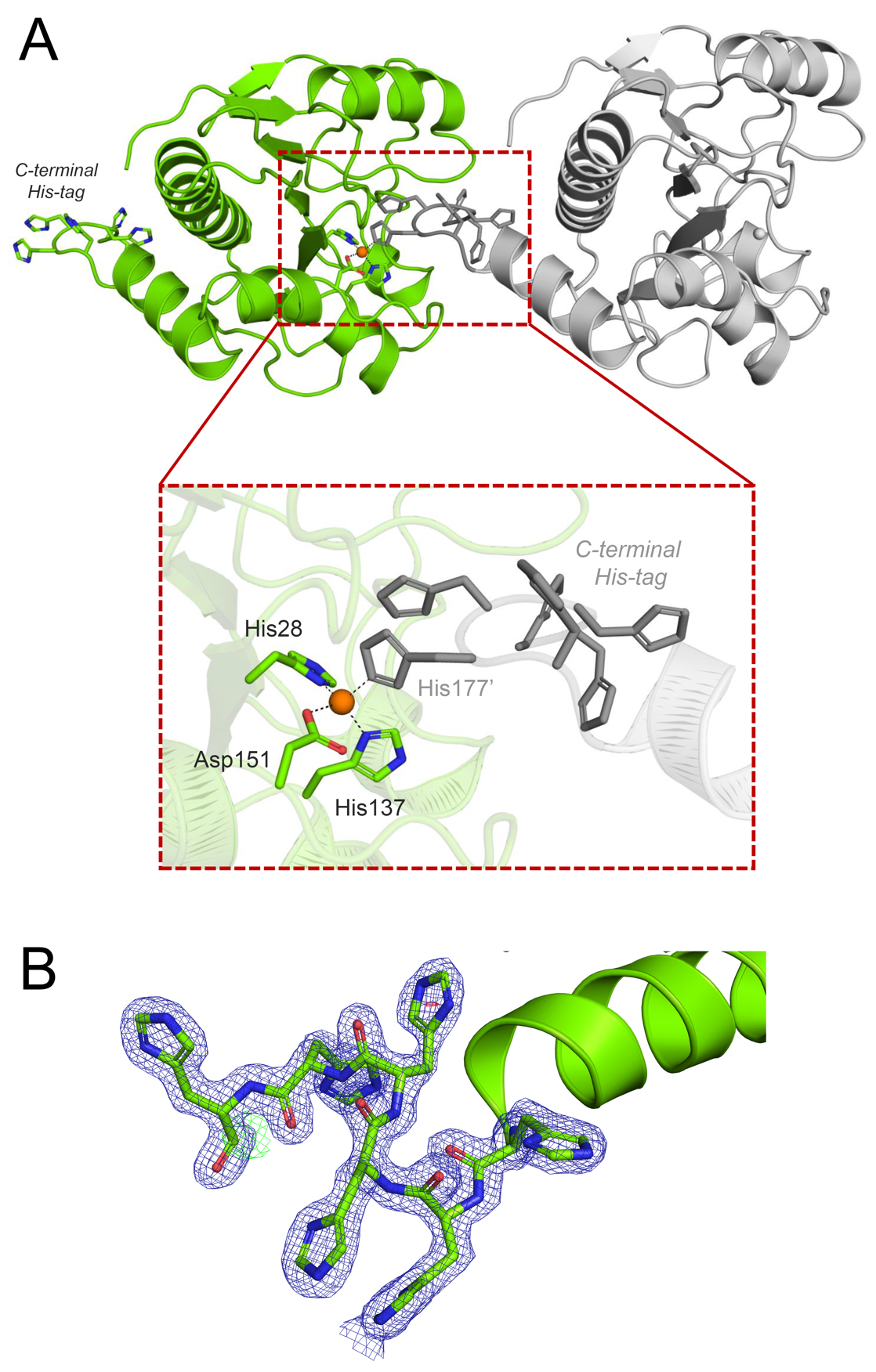
**

**Figure S4. Interaction between active site zinc ion and crystal contact. (A)** Top: Cartoon representation showing the PlyP100-D1 monomer (green) and a PlyP100-D1 monomer related by crystallographic symmetry (grey). The active site zinc ion is shown as an orange sphere. The histidine residues from the C-terminal His-tag of both monomers are shown as sticks. Bottom: Magnification of box highlighted in top panel. Hydrogen bond interactions are shown as black dashed lines. **(B)** Electron density quality of C-terminal His-tag. The 2*F*_o_-*F*_c_ map is contoured at 1.5 σ (blue) and the *F*_o_-*F*_c_ maps are contoured at +3.5 σ (green) and -3.5 σ (red). Figure produced with PyMOL (v.2.3.3, Schrödinger).

**
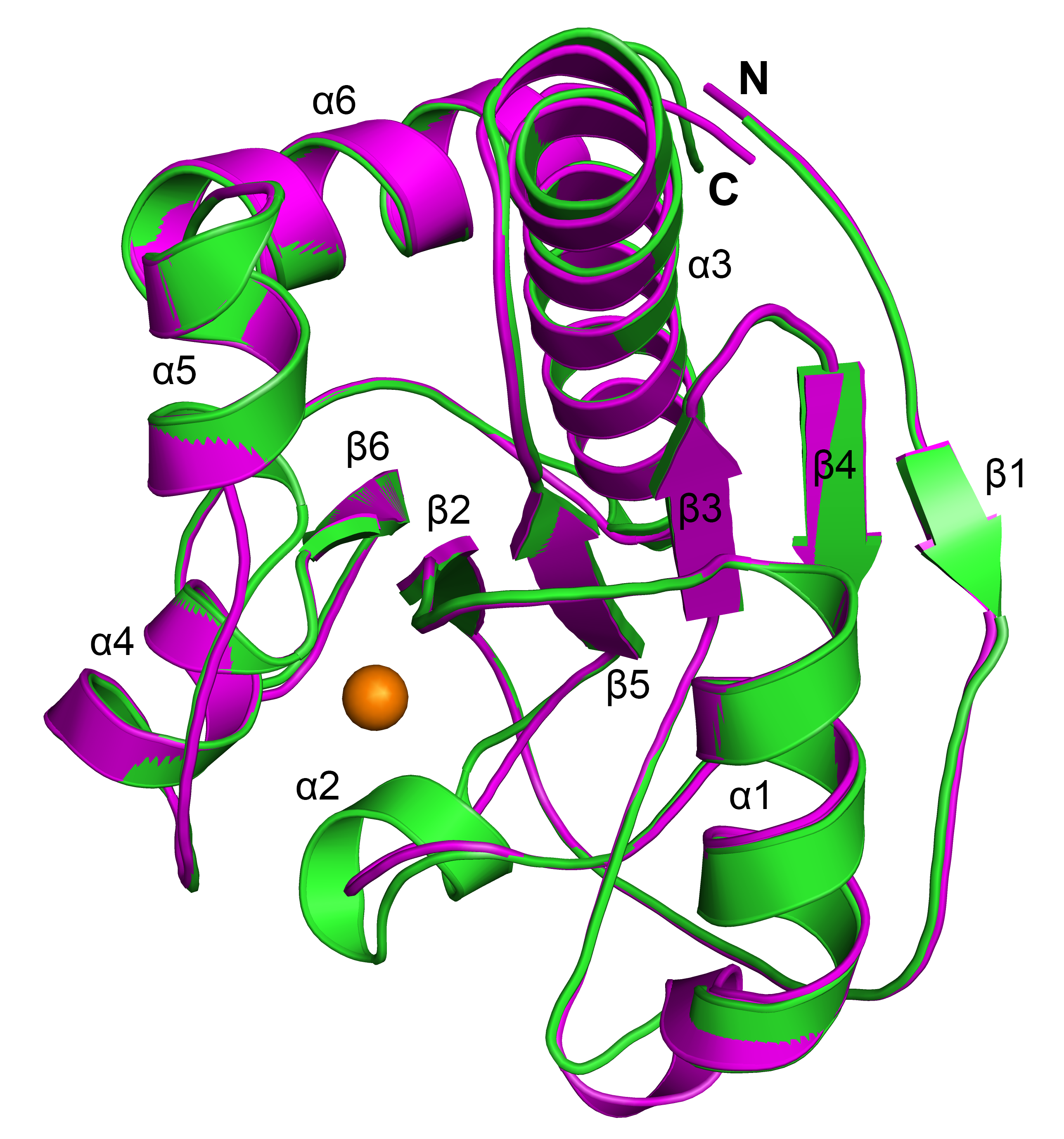
**

**Figure S5. Comparison of PlyP100-D1 domain with computationally generated model.** An Alphafold model was produced using the Alphafold webserver (<https://alphafold.ebi.ac.uk>) and the amino acid sequence of PlyP100 (residues 1-178, UniProt: Q30LD5) as the input. Individual monomers are shown as cartoon representations colored green (PlyP100-D1) or magenta (Alphafold model). Cα-atom superposition of the two structures is associated with an RMSD of 0.48 Å. Secondary structure elements including α-helices (α1-α6) and β-strands (β1-β6) are indicated. The catalytic zinc ion from the PlyP100-D1 X-ray crystal structure is shown as an orange sphere. Figure produced with PyMOL (version 3.0.4, Schrödinger).

*
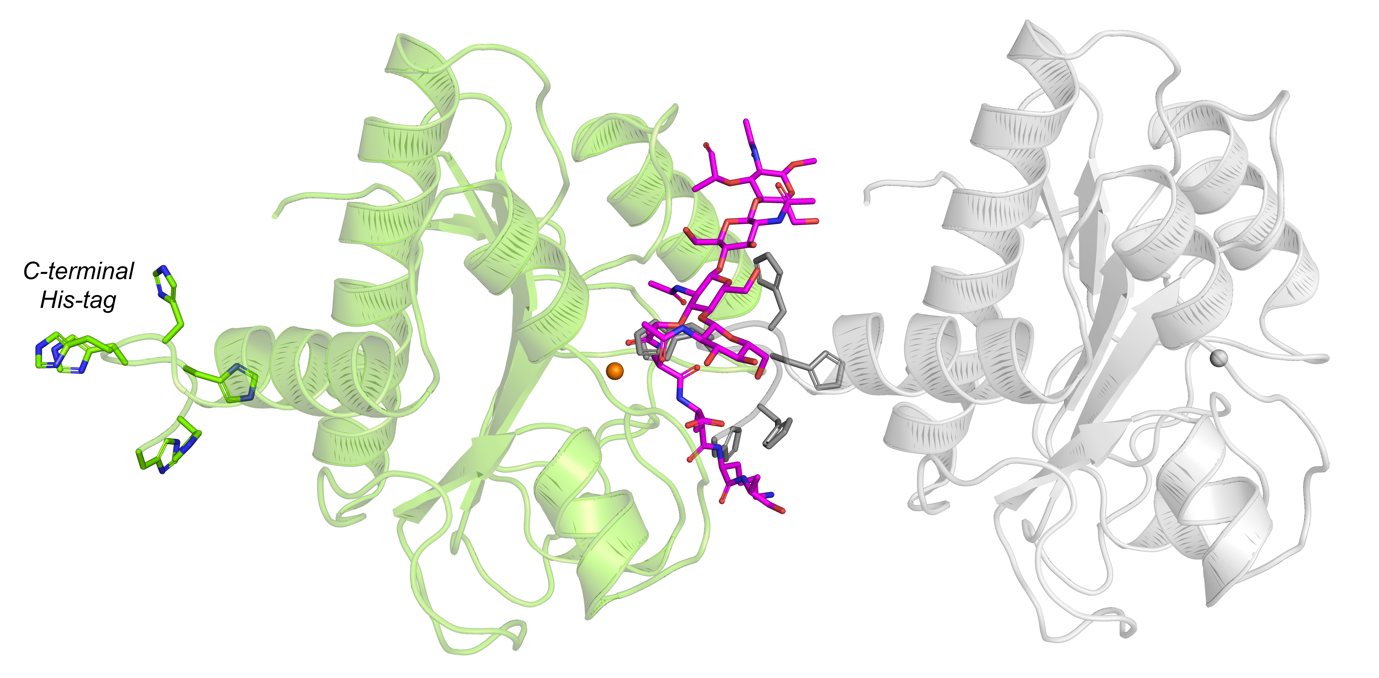
*

**Figure S6. Crystal contact His-tag is positioned in putative PlyP100-D1 active site.** Cartoon representation showing the PlyP100-D1 monomer (green) and a PlyP100-D1 monomer related by crystallographic symmetry (grey). PlyP100-D1 was superimposed with *S. pneumoniae* LytA (PDB ID: 5ctv) bound to an synthetic peptidoglycan fragment, which is displayed as a magenta stick model. The monomer of LytA is not shown for clarity. The active site zinc ion of PlyP100-D1 is shown as an orange sphere. The histidine residues from the C-terminal His-tag of both monomers are shown as sticks. Figure produced with PyMOL (v.2.3.3, Schrödinger).

**
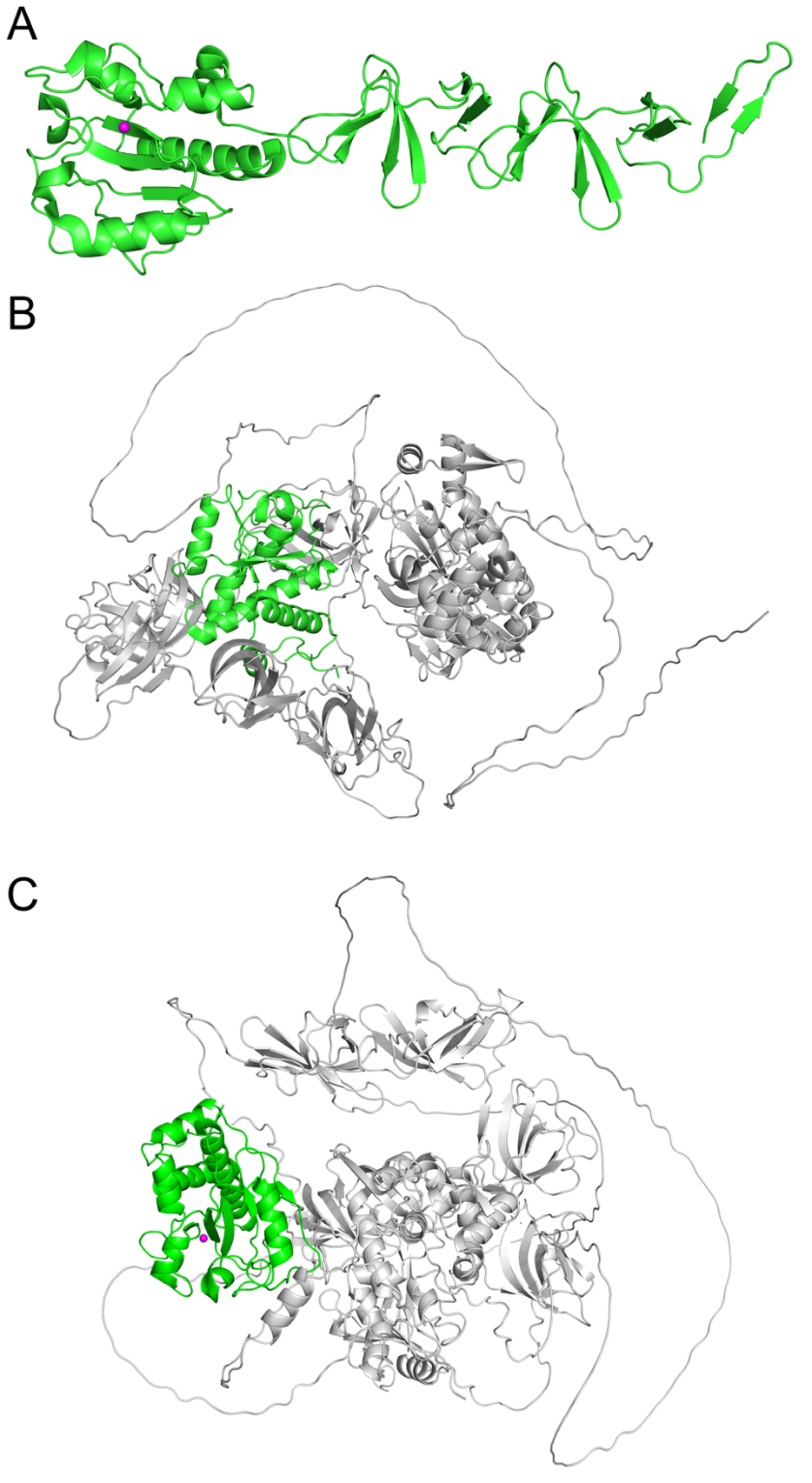
**

**Figure S7. Full length structures of structurally related amidases. (A)** Full-length structure of *S. pneumoniae* LytA (PDB ID: 4x3y) shown as a green cartoon. The catalytic zinc located in the catalytic domain (left) is shown as a magenta sphere. **(B)** *S. aureus* major autolysin structure (AtlA), highlighting the AmiA catalytic domain (green, PDB ID: 4knl). **(C)** *S. epidermidis* major autolysin structure (AtlE), highlighting the AmiE catalytic domain (green, PDB ID: 3lat). The catalytic zinc ion is shown as a magenta sphere. In panels **B-C** the predicted Alphafold models of full length AtlA and AtlE are shown in grey. Figure produced with PyMOL (v.2.3.3, Schrödinger).

**
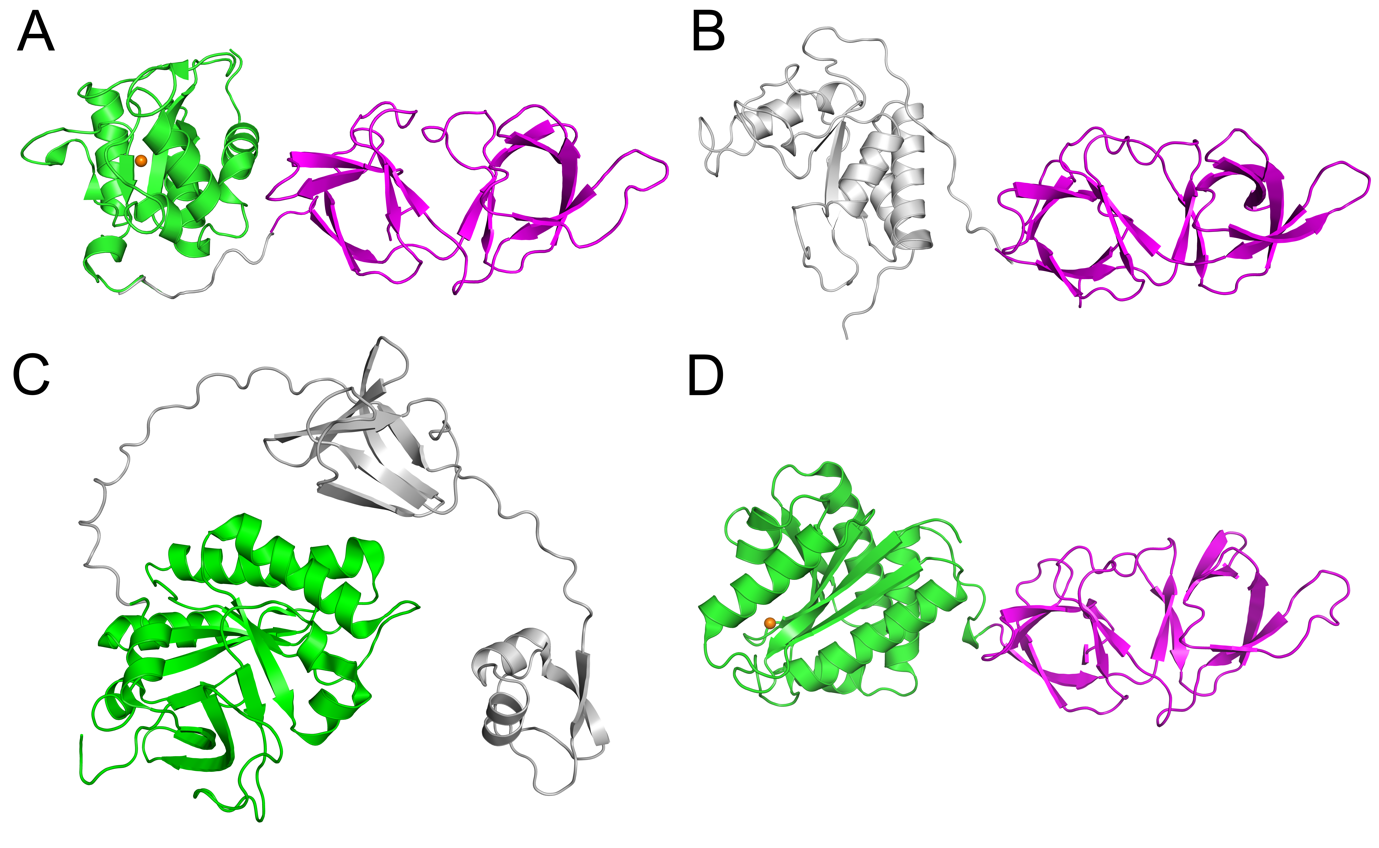
**

**Figure S8. Structures of Listeria phage endolysins in the Protein Data Bank (PDB).** Cartoon representations of proposed full length endolysin structures. **(A)** PlyA500: catalytic domain (green, PDB ID: 2vo9), cell wall binding domain (magenta, PDB ID: 6hx0). **(B)** PlyP35: cell wall binding domain (magenta, PDB ID: 6s4s). **(C)** PlyP40: catalytic domain (green, PDB ID: 4jz5). **(D)** PlyPSA: Full-length structure (PDB ID: 1xov) showing the catalytic domain (green) and cell wall binding domain (magenta). For endolysins in panels **A-C** the predicted Alphafold models (produced using the amino acids sequences associated with the UniProt IDs listed in **Table S5**) are shown in grey. Figure produced with PyMOL (v.2.3.3, Schrödinger).

**
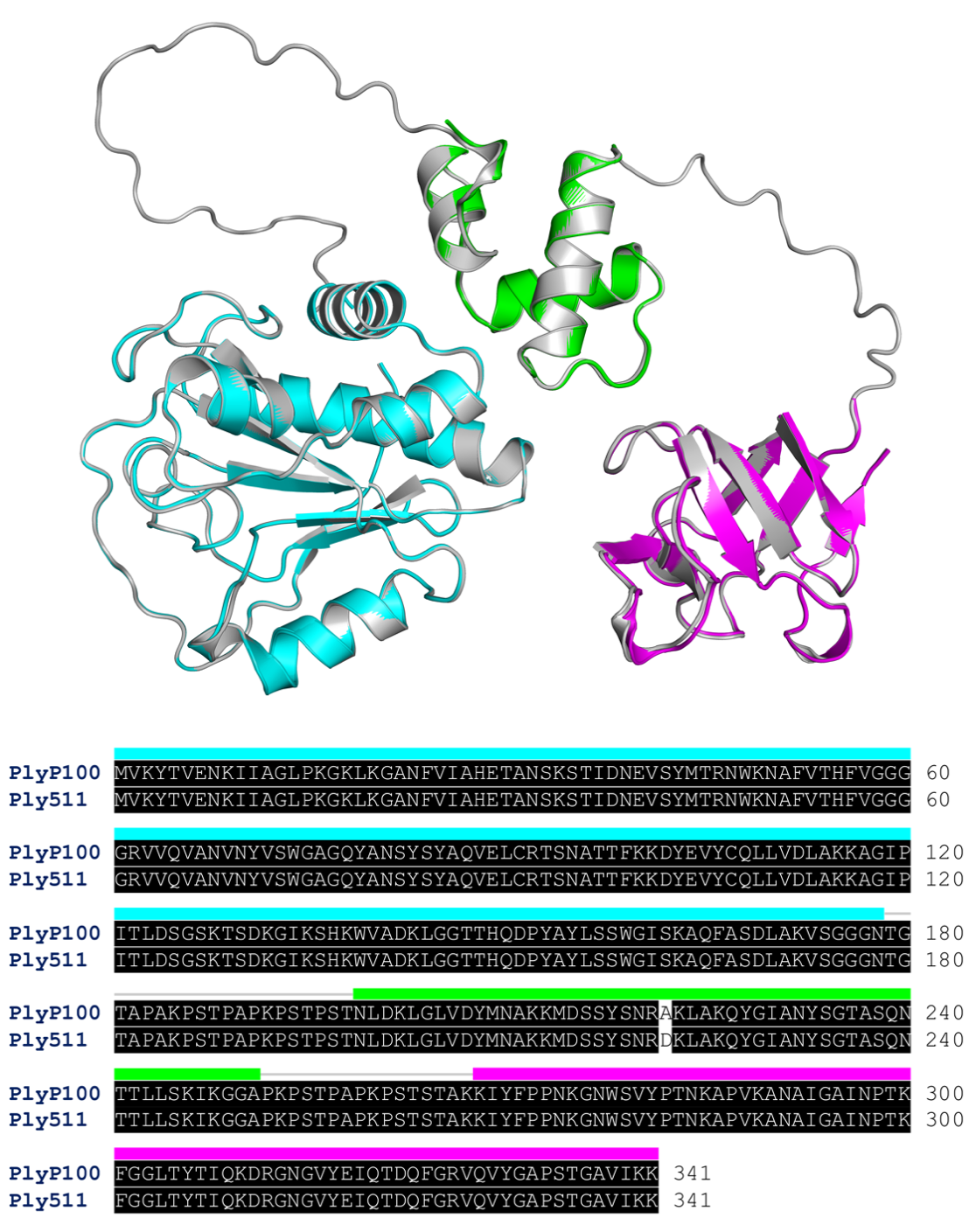
**

**Figure S9. Comparison of Ply511 and PlyP100 endolysins.** Top panel: Computational model of listeria phage endolysin Ply511 shown as a grey cartoon representation. The amino acid sequence of Ply118 (UniProt ID: Q38653) was used to generate the predicted structure using AlphaFold (4). Comparison of the Ply511 AlphaFold model with PlyP100-FL AlphaFold model (shown in **Figure 1**) indicated domains D1, D2 and D3 are structurally identical. As the orientation of the loops between the domains differ in the two AlphaFold structures, only the individual of PlyP100 are shown. The X-ray crystal structure of PlyP100-D1 is shown as a cyan cartoon, whereas the AlphaFold models of the PlyP100-D2 and D3 domains are shown as green and magenta cartoons, respectively. Bottom panel: Sequence alignment of PlyP100-FL (UniProt ID: Q30LD5) with Ply511. Identical residues are shaded black, while grey shading indicates amino acids with conserved physicochemical properties. The proposed domain structure of the PlyP100-FL AlphaFold model is shown above the alignment. The D1, D2, and D3 domains are coloured cyan, green and magenta, respectively. Figure produced with PyMOL (v.2.3.3, Schrödinger).

**Table S1. Top DALI search results for PlyP100-D1 AlphaFold model.**

| **Protein** | **Z-score** | **Rmsd (Å)** | **% id** | **PDB ID** | **Reference** |
| --- | --- | --- | --- | --- | --- |
| *S. pneumoniae* LytA | 27.8 | 1.5 | 38 | 4ivv | Mellroth *et al.,* 2014 (5) |
| *S. pneumoniae* LytA | 27.8 | 1.4 | 39 | 4x36 | Li *et al*., 2015 (6) |
| *S. pneumoniae* LytA | 27.7 | 1.4 | 38 | 5ctv | Sandalova et al., 2016 (7) |
| *S. aureus* AmiA | 25.0 | 1.5 | 29 | 4knl | Büttner *et al*., 2014 (8) |
| *S. epidermidis* AmiE | 24.8 | 1.6 | 29 | 3lat | Zoll *et al.,* 2010 (9) |

**Table S2. PlyP100 constructs used in this work.** Hexa-histidine tags are shown in blue.

| **Construct** | **Protein sequence** |
| --- | --- |
| PlyP100-FL | MVKYTVENKIIAGLPKGKLKGANFVIAHETANSKSTIDNEVSYMTRNWKNAFVTHFVGGGGRVVQVANVNYVSWGAGQYANSYSYAQVELCRTSNATTFKKDYEVYCQLLVDLAKKAGIPITLDSGSKTSDKGIKSHKWVADKLGGTTHQDPYAYLSSWGISKAQFASDLAKVSGGGNTGTAPAKPSTPAPKPSTPSTNLDKLGLVDYMNAKKMDSSYSNRAKLAKQYGIANYSGTASQNTTLLSKIKGGAPKPSTPAPKPSTSTAKKIYFPPNKGNWSVYPTNKAPVKANAIGAINPTKFGGLTYTIQKDRGNGVYEIQTDQFGRVQVYGAPSTGAVIKKLEHHHHHH |
| PlyP100-D1 | MVKYTVENKIIAGLPKGKLKGANFVIAHETANSKSTIDNEVSYMTRNWKNAFVTHFVGGGGRVVQVANVNYVSWGAGQYANSYSYAQVELCRTSNATTFKKDYEVYCQLLVDLAKKAGIPITLDSGSKTSDKGIKSHKWVADKLGGTTHQDPYAYLSSWGISKAQFASDLAKVSGGGNLEHHHHHH |
| PlyP100-D1 *for* *crystallography* | MVKYTVENKIIAGLPKGKLKGANFVIAHETANSKSTIDNEVSYMTRNWKNAFVTHFVGGGGRVVQVANVNYVSWGAGQYANSYSYAQVELCRTSNATTFKKDYEVYCQLLVDLAKKAGIPITLDSGSKTSDKGIKSHKWVADKLGGTTHQDPYAYLSSWGISKAQFASDLAKVSGHHHHHH |
| PlyP100-D1+2 | MVKYTVENKIIAGLPKGKLKGANFVIAHETANSKSTIDNEVSYMTRNWKNAFVTHFVGGGGRVVQVANVNYVSWGAGQYANSYSYAQVELCRTSNATTFKKDYEVYCQLLVDLAKKAGIPITLDSGSKTSDKGIKSHKWVADKLGGTTHQDPYAYLSSWGISKAQFASDLAKVSGGGNTGTAPAKPSTPAPKPSTPSTNLDKLGLVDYMNAKKMDSSYSNRAKLAKQYGIANYSGTASQNTTLLSKIKGGALEHHHHHH |
| PlyP100-D1+3 | MVKYTVENKIIAGLPKGKLKGANFVIAHETANSKSTIDNEVSYMTRNWKNAFVTHFVGGGGRVVQVANVNYVSWGAGQYANSYSYAQVELCRTSNATTFKKDYEVYCQLLVDLAKKAGIPITLDSGSKTSDKGIKSHKWVADKLGGTTHQDPYAYLSSWGISKAQFASDLAKVSGGGNTGTAPAKPSTPAPKPSTPSTPKPSTPAPKPSTSTAKKIYFPPNKGNWSVYPTNKAPVKANAIGAINPTKFGGLTYTIQKDRGNGVYEIQTDQFGRVQVYGAPSTGAVIKKLEHHHHHH |

**Table S3. Gram-positive bacterial strains used in this research.** PG: peptidoglycan. ATCC: <https://www.atcc.org>

| **Species** | **Strain** | **PG type** | **Serotype** | **Isolation source** |
| --- | --- | --- | --- | --- |
| *Listeria monocytogenes* | NRRL B-33419 | A1𝛄 | 1/2a | Sliced turkey outbreak (10) |
| *Listeria monocytogenes* | NRRL B-33424 | A1𝛄 | 1/2b | Chocolate milk outbreak (10) |
| *Listeria monocytogenes* | NRRL B-33420 | A1𝛄 | 4b | RTE meat outbreak (10) |
| *Listeria monocytogenes* | NRRL B-33513 | A1𝛄 | 4b | Pate outbreak (10) |
| *Listeria monocytogenes* | NRRL B-33104 | A1𝛄 | 4b | Jalisco cheese outbreak (10) |
| *Listeria innocua* | ATCC 33090 | A1𝛄 | 6a | Cow brain (ATCC) |
| *Lactobacillus plantarum* | ATCC 8014 | A1𝛄 |  | Corn silage and/or sauerkraut (ATCC, (11)) |
| *Brevibacterium linens* | ATCC 9172 | A1𝛄 |  | Harzerkase cheese (ATCC) |
| *Aerococcus viridans* | ATCC 11563 | A1α |  | Air sample (ATCC) |
| *Leuconostoc mesenteroides* subsp. *mesenteroides* | ATCC 8293 | A3α |  | Fermenting olives (ATCC) |
| *Lactobacillus fermentum* | ATCC 14931 | A4β |  | Fermented beets (ATCC) |

**Table S4. Top DALI search results for PlyP100-D1 X-ray structure.**

| **Protein** | **Z-score** | **Rmsd (Å)** | **% id** | **PDB ID** | **Details** | **Reference** |
| --- | --- | --- | --- | --- | --- | --- |
| *S. pneumoniae* LytA | 27.6 | 1.3 | 39 | 4x36 | Full structure | Li *et al*., 2015 (6) |
| *S. pneumoniae* LytA | 27.6 | 1.5 | 38 | 4ivv | Catalytic domain | Mellroth *et al.,* 2014 (5) |
| *S. pneumoniae* LytA | 27.4 | 1.4 | 38 | 5ctv | Catalytic domain | Sandalova et al., 2016 (7) |
| *S. aureus* AmiA | 25.4 | 1.8 | 29 | 4knl | Catalytic domain | Büttner *et al*., 2014 (8) |
| *S. epidermidis* AmiE | 24.9 | 1.8 | 29 | 3lat | Catalytic domain | Zoll *et al.,* 2010 (9) |

**Table S5. Listeria phage endolysin structures in the Protein Data Bank (PDB)**

| **Protein** | **PDB ID** | **UniProt** | **Details** | **Reference** |
| --- | --- | --- | --- | --- |
| PlyA500 | 6hx0 | Q37979 | Cell wall binding domain | Shen et al., 2021 (12) |
| PlyA500 | 2vo9 | Q37979 | Catalytic domain | [Korndörfer](https://scripts.iucr.org/cgi-bin/citedin?search_on=name&author_name=Kornd%26%23246;rfer,%20I.P.) et al., 2008 (13) |
| PlyP35 | 6s4s | A8ATR6 | Cell wall binding domain | Unpublished |
| PlyP35 | 6s3y | A8ATR6 | Cell wall binding domain | Unpublished |
| PlyP35 | 6thj | A8ATR6 | Cell wall binding domain | Unpublished |
| PlyP40 | 4jz5 | B6D7J9 | Catalytic domain | Unpublished |
| PlyPSA | 1xov | Q8W5Y8 | Full structure | [Korndörfer](https://scripts.iucr.org/cgi-bin/citedin?search_on=name&author_name=Kornd%26%23246;rfer,%20I.P.) et al., 2006 (14) |

**Table S6. X-ray crystallography data collection and refinement statistics**

|  | **PlyP100 catalytic domain** |
| --- | --- |
| **Data collection** |  |
| PDB code | 9HTU |
| Space group | P2_1_2_1_2_1_ |
| Cell dimensions: |  |
| a, b, c (Å) | 37.3, 65.8, 96.5 |
| α, β, γ (°) | 90.0, 90.0, 90.0 |
| Resolution (Å) | 1.80-96.5 (1.80-1.84) |
| Total reflections | 272280 (9077) |
| Unique reflections | 22719 (1239) |
| *R*_merge_ | 0.140 (0.739) |
| *R*_pim_ | 0.059 (0.416) |
| CC_1/2_ | 0.978 (0.798) |
| *I/*σ | 10.8 (2.2) |
| Completeness | 99.6 (95.0) |
| Redundancy | 12.0 (7.3) |
| **Refinement** |  |
| *R*_work_/*R*_free_ (%) | 15.2/18.3 |
| *B-*factors: |  |
| Protein | 18.3 |
| Ligand | 38.5 |
| Ion | 16.6 |
| Water | 32.3 |
| R.m.s. deviations: |  |
| Bond lengths (Å) | 0.013 |
| Bond angles (°) | 1.72 |
| Ramachandran statistics: |  |
| Favoured (%) | 100 |
| Outliers (%) | 0 |

Values in parentheses are for the highest-resolution shell.

**References**

1. Neelamegham, S., Aoki-Kinoshita, K., Bolton, E., Frank, M., Lisacek, F., Lütteke, T. *et al.* (2019) Updates to the Symbol Nomenclature for Glycans guidelines *Glycobiology* **29**, 620-624

2. Tsuchiya, S., Aoki, N. P., Shinmachi, D., Matsubara, M., Yamada, I., Aoki-Kinoshita, K. F. *et al.* (2017) Implementation of GlycanBuilder to draw a wide variety of ambiguous glycans *Carbohydr Res* **445**, 104-116

3. Dortet, L., Radoshevich, L., Veiga, E., and Cossart, P. (2019) Listeria monocytogenes☆ In *Encyclopedia of Microbiology (Fourth Edition)*, Schmidt TM, ed. Academic Press, Oxford 803-818

4. Jumper, J., Evans, R., Pritzel, A., Green, T., Figurnov, M., Ronneberger, O. *et al.* (2021) Highly accurate protein structure prediction with AlphaFold *Nature* **596**, 583-589

5. Mellroth, P., Sandalova, T., Kikhney, A., Vilaplana, F., Hesek, D., Lee, M. *et al.* (2014) Structural and Functional Insights into Peptidoglycan Access for the Lytic Amidase LytA of Streptococcus pneumoniae *mBio* **5**, 10.1128/mbio.01120-01113

6. Li, Q., Cheng, W., Morlot, C., Bai, X.-H., Jiang, Y.-L., Wang, W. *et al.* (2015) Full-length structure of the major autolysin LytA *Acta Crystallographica Section D* **71**, 1373-1381

7. Sandalova, T., Lee, M., Henriques-Normark, B., Hesek, D., Mobashery, S., Mellroth, P. *et al.* (2016) The crystal structure of the major pneumococcal autolysin LytA in complex with a large peptidoglycan fragment reveals the pivotal role of glycans for lytic activity *Molecular Microbiology* **101**, 954-967

8. Büttner, F. M., Zoll, S., Nega, M., Götz, F., and Stehle, T. (2014) Structure-Function Analysis of Staphylococcus aureus Amidase Reveals the Determinants of Peptidoglycan Recognition and Cleavage *Journal of Biological Chemistry* **289**, 11083-11094

9. Zoll, S., Pätzold, B., Schlag, M., Götz, F., Kalbacher, H., and Stehle, T. (2010) Structural Basis of Cell Wall Cleavage by a Staphylococcal Autolysin *PLOS Pathogens* **6**, e1000807

10. Ibarra-Sánchez, L. A., Van Tassell, M. L., and Miller, M. J. (2018) Antimicrobial behavior of phage endolysin PlyP100 and its synergy with nisin to control Listeria monocytogenes in Queso Fresco *Food Microbiology* **72**, 128-134

11. Fred, E. B., Peterson, W. H., and Anderson, J. A. (1921) THE CHARACTERISTICS OF CERTAIN PENTOSE-DESTORYING BACTERIA, ESPECIALLY AS CONCERNS THEIR ACTION ON ARABINOSE AND XYLOSE *Journal of Biological Chemistry* **48**, 385-412

12. Shen, Y., Kalograiaki, I., Prunotto, A., Dunne, M., Boulos, S., Taylor, N. M. I. *et al.* (2021) Structural basis for recognition of bacterial cell wall teichoic acid by pseudo-symmetric SH3b-like repeats of a viral peptidoglycan hydrolase *Chemical Science* **12**, 576-589

13. Korndorfer, I. P., Kanitz, A., Danzer, J., Zimmer, M., Loessner, M. J., and Skerra, A. (2008) Structural analysis of the l-alanoyl-d-glutamate endopeptidase domain of Listeria bacteriophage endolysin Ply500 reveals a new member of the LAS peptidase family *Acta Crystallographica Section D* **64**, 644-650

14. Korndörfer, I. P., Danzer, J., Schmelcher, M., Zimmer, M., Skerra, A., and Loessner, M. J. (2006) The Crystal Structure of the Bacteriophage PSA Endolysin Reveals a Unique Fold Responsible for Specific Recognition of Listeria Cell Walls *Journal of Molecular Biology* **364**, 678-689
